# Theoretical models applied to understand infection prevention and control practices of healthcare workers during the COVID-19 pandemic: A systematic review

**DOI:** 10.1177/17571774241251645

**Published:** 2024-05-16

**Authors:** Deepti KC, Jan Smith, Kay Currie, Valerie Ness

**Affiliations:** Safeguarding Health through Infection Prevention (SHIP) Research Group, School of Health and Life Sciences, Glasgow Caledonian University, Glasgow, UK

**Keywords:** Behaviour change theory, infection prevention and control, healthcare workers, COVID-19, systematic review

## Abstract

**Background:**

Effective infection prevention and control (IPC) practices among healthcare workers are crucial to prevent the spread of COVID-19 and other infections in healthcare settings.

**Aim:**

To synthesise evidence on behaviour change theories, models, or frameworks applied to understand healthcare workers’ IPC practices during the COVID-19 pandemic.

**Methods:**

PubMed, EBSCOhost interface, ProQuest interface, MEDLINE (Ovid), and grey literature were searched for primary studies published between December 2019 and May 2023. The Mixed Method Appraisal Tool evaluated the methodological quality of the studies. Two reviewers independently completed study selection, data extraction, and quality assessment.

**Results:**

The search yielded 2110 studies, of which 19 were included. Seven behaviour change theories, models, and frameworks were identified, with the Health Belief Model and Theoretical Domains Framework being the most employed. Based on these theories, models, and frameworks, the included studies identified cognitive, environmental, and social factors influencing healthcare workers’ compliance with COVID-19 IPC practices.

**Discussion:**

This review offers insights into the critical role of behavioural change theories, models, or frameworks in understanding the factors influencing healthcare workers’ compliance with IPC practices during COVID-19. It also highlights the potential of these theories in guiding the development of evidence-based interventions to improve healthcare workers’ compliance with IPC practices.

## Background

The healthcare transmission of SARS-CoV-2, the virus responsible for coronavirus disease (COVID-19), has been a significant concern throughout the pandemic. Nevertheless, infection prevention and control (IPC) practices, like hand hygiene, respiratory hygiene, and environmental cleanliness, can prevent healthcare transmission of COVID-19 and other avoidable infections (World Health Organization, 2021).

However, despite the guidelines set by the World Health Organization (WHO) during COVID-19, studies have shown that healthcare workers (HCWs) have inadequate adherence to recommended IPC practices (Abed Alah et al., 2021; Bashirian et al., 2020). The challenges go beyond the lack of personal protective equipment (PPE) and hand-washing agents (Abed Alah et al., 2021). Non-adherence has also been linked to HCWs’ attitudes towards COVID-19 and perceived difficulties in adopting IPC measures (Smith et al., 2021). It is imperative to address the individual, organisational, and environmental challenges that impede HCWs’ motivation and capability to adhere to IPC practices, as Houghton et al. (2020) highlighted in their review. Furthermore, tailored interventions are necessary to overcome these challenges and support HCWs in implementing and sustaining effective IPC practices.

Applying behaviour change theory is critical in exploring evidence-based practices, particularly addressing poor staff performance (Greene and Wilson, 2022). This approach enables the identification of critical determinants of behaviour and assists in developing implementation strategies by providing a theoretical foundation for understanding the physical and psychological processes expected to regulate behaviour (Michie et al., 2005). However, numerous theories, models, or frameworks of behaviour change exist. For example, Michie et al. (2014), in their book, described 83 behaviour change theories across the behavioural and social sciences. Additionally, theories, models, and frameworks are distinct concepts. While behaviour change theories help explain why behaviours change over time and how they can be changed (Darnton, 2008), models and frameworks do not define the change mechanism. Instead, they aid in understanding specific behaviours by identifying the underlying factors that drive them (Nilsen, 2015).

The COVID-19 pandemic highlighted the critical need for systematic use of behaviour change theory to identify factors influencing behaviour change, understand the change process, and inform intervention development (West et al., 2020). The plethora of available theories, models, and frameworks, each with its unique focus and scope, also necessitates a systematic examination to determine their applicability and effectiveness in understanding and improving IPC practices of HCWs. By understanding how these theories have been applied in COVID-19 studies, researchers can identify the determinants influencing the IPC behaviour of HCWs, which in turn can inform policy and help develop resilience and new behavioural patterns to prepare for future pandemics (West et al., 2020).

Therefore, this systematic review aims to synthesise evidence on which theories, models, or frameworks of behaviour change have been used to understand the IPC practices of HCWs in the context of COVID-19. Moreover, this review examines the factors influencing HCWs’ compliance with COVID-19 IPC practices, drawing insights from the theories, models, and frameworks of behaviour change.

## Methods

### Review design

This review followed the Preferred Reporting Items for Systematic Reviews and Meta-Analysis (PRISMA) guidelines (Page et al., 2021) to provide a transparent and comprehensive summary of the studies (Supplemental File 1). The protocol for this review was registered in the PROSPERO database (CRD42022332006).

### Eligibility criteria

Inclusion criteria were studies published in English between December 2019 and March 2023. Studies needed to report the application of a behaviour change theory, model, or framework to examine HCWs’ IPC practices like hand hygiene and droplet isolation practices, including the use of masks, gloves, goggles, and gowns, which align with the COVID-19 guidelines set by the WHO (World Health Organization, 2021). Primary studies of any design (qualitative, quantitative, or mixed) were considered to locate a broad range of studies.

Exclusion criteria were studies focusing on a mixed sample where outcomes related to HCWs could not be separated from other populations (e.g., administrative staff and the general public). Studies published in languages other than English and non-primary studies, such as systematic reviews and conference proceedings, were also excluded.

### Search strategy

A search was conducted on July 30th, 2022, and updated on May 30th, 2023, using four databases: EBSCOhost interface, ProQuest interface, PubMed, and MEDLINE (Ovid), chosen for their wide subject and content-specific coverage. The search encompassed all 15 databases on the EBSCOhost interface, including AMED and CINAHL Plus with Full Text, and all 18 databases on the ProQuest interface, including APA PsycInfo, Coronavirus Research Database, and Psychology Database. The search was conducted using index terms and free-text key terms within the titles and abstracts, focusing on four domains: behaviour change theories, infection prevention and control, healthcare workers, and COVID-19. The search strategy was tailored to each database’s functions for comprehensiveness. The search was limited to papers published from December 2019 to March 2023, corresponding to the emergence of COVID-19 in 2019. A language limiter was applied due to translation constraints. The first author created the search strategy, reviewed and approved by the research team (Supplemental File 2 for detailed search results).

A Google Scholar search was also conducted to locate grey literature to reduce selection bias and enhance the review’s comprehensiveness (Paez, 2017). Furthermore, the reference lists of potential papers were manually scanned to identify papers not indexed in the selected databases. Search results were exported to RefWorks, duplicates were removed, and the remaining records were screened against the eligibility criteria.

### Study selection

Two reviewers independently screened the titles and abstracts of search results against the eligibility criteria. Relevant articles and those with insufficient evidence were selected for full-text review to determine eligibility. Disagreements between the reviewers were resolved through discussion or involvement of a third reviewer. The third reviewer confirmed the eligibility of all included studies.

### Data extraction and quality assessment

Details on study authors, publication year, country of origin, settings, study design, sample, underpinning theories, study outcomes, and key findings were extracted in a Microsoft Excel spreadsheet. The Mixed Method Appraisal Tool (MMAT) was used to appraise the studies, as it is designed to address the challenges of critical appraisal in systematic mixed studies reviews (Hong et al., 2018). All studies met the review’s aim, so none were excluded based on quality.

### Data synthesis

The review’s findings were described using a formal narrative synthesis approach (Popay et al., 2006). Studies were grouped per theories, models, or frameworks of behaviour change, and outcomes were tabulated and synthesised narratively.

## Results

**Figure 1.**
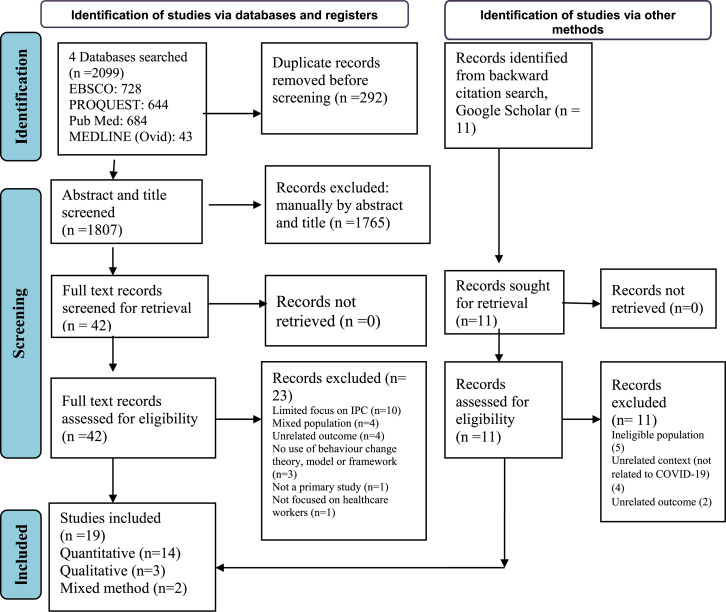
PRISMA flow diagram showing selection of studies for inclusion in the systematic review.

As shown in the PRISMA flow diagram (Figure 1), the database search identified 2099 records, and 11 were found in additional sources. After removing duplicates (*n* = 292), 1807 records underwent title and abstract screening. The full texts of 42 records from the database search and 11 from additional sources were retrieved and evaluated against the review’s eligibility criteria. Finally, 34 records were excluded (Supplemental File 3), and 19 were included in this review.

### Characteristics of included studies

Table 1 summarises the characteristics of the included studies. This review included 19 studies representing 15 different countries: Australia (*n* = 1), Bangladesh (*n* = 1), Canada (*n* = 1), China (*n* = 1), Finland (*n* = 1), Germany (*n* = 2), Hong Kong (*n* = 1), India (*n* = 1), Iran (*n* = 2), Korea (*n* = 2), Saudi Arabia (*n* = 1), Thailand (*n* = 1), Turkey (*n* = 1), the United States (*n* = 1), and the United Kingdom (*n* = 1). One study (Van Hout et al., 2022) included participants from 40 European countries. Among these studies, 14 were quantitative, three were qualitative, and two used mixed methods. Most studies (*n* = 14) involved mixed groups of HCWs’, while some focused on specific groups like nurses (*n* = 4) and dentists (*n* = 1).

**Table 1.** Characteristics of included studies (ordered alphabetically).

Author	Country	Study design	Participants/settings	Data collection tool/method	Theory used	Targeted IPC practices	Outcome measures
Bashirian et al. (2020)	Iran	Quantitative, cross-sectional analytical	761 HCWs from five University Teaching hospitals	Self-reported questionnaire	PMT	COVID-19 IPC practices specified as hand hygiene and glove and mask use	Compliance and predictors
Castro-Sánchez et al. (2021)	UK	Quantitative, evaluative	261 hospital staff	Paper and electronic survey distributed via email	TDF and COM-B	PPE use	Evaluation of PPE Helper program
Curtis et al. (2022)	Australia	Qualitative	280 hospital emergency department staff	Direct observation	TDF and BCW	PPE use	Barriers to PPE use and evaluation of PPE intervention
Derksen et al. (2020)	Germany	Quantitative, observational	115 HCWs from two obstetric university hospitals	Observation and self-reported questionnaire (paper and pencil questionnaire)	HAPA	Hand hygiene	Adherence and determinants
Jeong and Eun (2023)	Korea	Quantitative, cross-sectional	143 nurses	Self-administered questionnaire	HBM	COVID-19 IPC practices (unspecified)	Levels and associated factors
Kim and Kim (2023)	Korea	Quantitative, cross-sectional	161 emergency nurses from five tertiary and general hospitals	Self-administered questionnaire	HBM	COVID-19 IPC practices (unspecified)	Associated factors
Limkunakul et al. (2022)	Thailand	Quantitative, cross-sectional	273 HCWs from a university-affiliated medical centre	Self-administered questionnaire	HBM	COVID-19 IPC practices specified as hand hygiene, surgical masks use, and disinfection	Predictors
Lohiniva et al. (2022)	Finland	Mixed-method study	422 HCWs in LTCFs	Web-based survey followed by telephone-based in-depth interviews	TDF	COVID-19 IPC practices specified as hand hygiene, PPE use, and environmental cleaning	Compliance and factors
Mortada et al. (2021)	Saudi Arabia	Quantitative, cross-sectional survey	385 hospital emergency department HCWs	Online survey circulated via social media like Facebook, Twitter, and WhatsApp	PMT	COVID-19 IPC practices (unspecified)	Behavioural intention and determinants
Müller et al. (2021)	Germany	Qualitative	12 dentists practicing in dental offices	Telephone interviews	TDF and BCW	COVID-19 IPC practices specified as PPE use, hand hygiene, and disinfection	Barriers and enablers
Salwa et al. (2022)	Bangladesh	Explanatory sequential mixed-method study	604 physicians and nurses from tertiary care facilities	Self-administered questionnaire	HBM	COVID-19 IPC practices specified as disinfection, decontamination, hand hygiene, and PPE use	Compliance and associated factors
Seitz et al. (2021)	USA	Quantitative, cross-sectional survey	308 hospital emergency department HCWs	Online survey distributed via email	TPB	PPE use	Knowledge, attitude, and practice
Silverberg et al. (2021)	Canada	Quantitative, cross-sectional survey	319 nurses from academic and community hospitals	Survey using REDCap electronic data capture tools	TDF	COVID-19 IPC practices specified as hand hygiene, use of mask, gloves, gowns and face shields, and PPE use	Adherence and associated barriers and facilitators
Sin and Rochelle (2022)	Hong Kong	Quantitative, cross-sectional survey	122 nurses from public hospitals	Online survey	TPB	Hand hygiene	Beliefs and behaviours
Sivaraman (2022)	India	Qualitative, phenomenological	15 HCWs from department of otorhinolaryngology of a tertiary care teaching hospital	Interviews	HBM	PPE use	Behavioural intention
Toghanian et al. (2022)	Iran	Quantitative, cross-sectional	270 HCWs from academic/educational hospitals	Self-administered questionnaire	PMT	COVID-19 IPC practices (unspecified)	Related factors
Türktemiz et al. (2021)	Turkey	Quantitative, cross-sectional survey	216 HCWs from hospitals	Online survey	HBM	PPE use	Perceptions and attitude
van Hout et al. (2022)	European countries	Quantitative, cross-sectional survey	2289 HCWs from hospitals	Online surveys distributed via electronic data capturing (EDC) systems Castor v2020.1.9 and Qualtrics survey platform (Provo, Utah)	TDF	COVID-19 IPC practices specified as hand hygiene and PPE use	Perceptions and compliance
Yang et al. (2021)	China	Quantitative, cross-sectional survey	768 HCWS from tertiary public hospitals	Self-administered questionnaire	TDF	COVID-19 IPC practices Specified as hand hygiene, overall droplet isolation, and google and gown use	Levels and determinants

BCW: Behaviour Change Wheel; COM-B: capability, opportunity, motivation and behaviour; HAPA: Health Action Process Approach; HBM: Health Belief Model; HCWs: health care workers; IPC: infection prevention and control; LTCFs: long-term care facilities; PMT: Protection Motivation Theory; PPE: personal protective equipment; TDF: Theoretical Domain Framework; TPB: Theory of Planned Behaviour.

### Behavioural theories, models, or frameworks and targeted behaviours

This review identified seven behaviour change theories, models, and frameworks (detailed in Supplemental File 4) used to explore the IPC practices of HCWs during COVID-19. These included the Health Belief Model, Theoretical Domains Framework, Protection Motivation Theory, Theory of Planned Behaviour, Health Action Process Approach model, Behaviour Change Wheel, and the Capability Opportunity Motivation-Behaviour (COM-B) model. Twelve studies examined multiple IPC practices, such as hand hygiene, PPE usage, and disinfecting and decontamination practices. Five studies focused on PPE usage and two on the hand hygiene behaviour of HCWs.

### Results of individual studies

The study results are presented narratively below according to specific behaviour change theories, models, and frameworks (detailed in Supplemental File 4) and then sub-divided into targeted IPC practices. Additional results are included in Supplemental File 5.

### Health Belief Model (HBM)

The HBM (Rosenstock, 1974) seeks to predict health-related behaviour through key belief patterns, including perceived susceptibility, perceived severity, perceived benefits, perceived barriers, cues to action, and self-efficacy. Six included studies utilised the HBM, with four examining multiple IPC practices and two PPE usage.

#### Multiple IPC practices

Jeong and Eun (2023) and Kim and Kim (2023) used Likert scale surveys based on the HBM to explore nurses’ COVID-19 IPC practices. Jeong and Eun (2023) highlighted the importance of perceived susceptibility, indicating that nurses who see themselves as more susceptible to COVID-19 were more likely to adopt IPC practices. Kim and Kim (2023) found that nurses’ perceptions of COVID-19 severity positively influenced their IPC practices. However, perceived barriers, such as the inconvenience of using PPE, made it more challenging to implement IPC practices. These studies showed that nurses’ beliefs regarding susceptibility to COVID-19 and its severity influenced their IPC practices.

Limkunakul et al. (2022) and Salwa et al. (2022) used HBM-based Likert scale surveys to identify factors associated with preventive behaviours among HCWs. Limkunakul et al. (2022) found significant factors associated with preventive behaviours, such as perception of COVID-19 risk, barriers to prevention strategies, benefits of following preventive practices, and perception of personal preventability (*p* < .001). Salwa et al. (2022) found positive associations between perceived benefits (benefits of practising IPC measures), self-efficacy (belief in the ability to perform IPC practices), cues to action (trust in the administration, policymakers, and government in taking appropriate measures), and compliance with IPC guidance. This study also found that perceived barriers to compliance included PPE unavailability, workplace insecurity, and unreliable information sources. These results highlight that promoting positive perceptions of IPC measures and enhancing HCWs’ self-efficacy can improve compliance with IPC practice.

#### Personal protective equipment usage

Sivaraman et al. (2022) and Türktemiz et al. (2021) explored HCWs’ perspectives on and intentions to use PPE. Sivaraman et al. (2022) utilised an HBM-based interview guide and found that perceived severity and susceptibility of COVID-19, perceived barriers (physical discomfort and communication challenges), perceived benefits, self-efficacy (familiarity with PPE use), and cues to action (timely release of updated guidelines) influenced PPE usage among HCWs. Meanwhile, Türktemiz et al. (2021), using an HBM-based survey, found that HCWs held positive beliefs about the effectiveness of PPE (perceived benefits) but viewed PPE use as a complex process constraining their professional skills (perceived barriers). These studies collectively highlight that efforts to boost PPE use among HCWs should target perceived barriers, highlight its benefits, promote self-efficacy, and provide clear guidelines.

### Theoretical Domains Framework (TDF)

The TDF is a comprehensive framework integrating components from 33 behaviour change theories into 14 domains (originally 12) (Cane et al., 2012; Michie et al., 2005). Four studies utilised the TDF focusing on multiple IPC practices of HCWs.

#### Multiple IPC practices

Lohiniva et al. (2022) used a mixed-method approach, combining surveys and qualitative interviews, both based on TDF. This study identified three TDF domains significantly influencing nurses’ compliance with IPC practices: environmental context and resources (changes in professional duties and lack of staff planning for emergencies), reinforcement (management culture and physical absence of management), and beliefs about capabilities (knowledge of applying IPC measures, nature of tasks, and infrastructure).

Silverberg et al. (2021) utilised a survey developed by WHO where TDF domains were used to ensure the survey items’ comprehensiveness. Findings revealed that nurses strongly believed in the effectiveness of IPC practices, with social and professional role playing a significant role in compliance. Likewise, Van Hout et al. (2022) used a survey based on the WHO protocol and TDF domains. Positive beliefs towards the protective effects of IPC practices were reported by 79% of HCWs, and positive social influences, such as colleagues adhering to IPC practices and encouragement from senior staff, facilitated compliance.

Yang et al. (2021), using a TDF-based survey, investigated determinants of hand hygiene and droplet isolation behaviours of HCWs. Results revealed the environmental context and resource (availability of PPE and hand hygiene facilities) domain as significantly related to hand hygiene and droplet isolation behaviours. Knowledge domain (lack of knowledge) was significantly associated with lower compliance with goggles and gown use. Emotion (fear, shame, and guilt of not following guidelines) and social influences (pressure from colleagues and department leaders) were significantly associated with droplet isolation behaviours.

These studies collectively highlighted the role of environmental context, knowledge, and social factors in influencing HCWs’ IPC practices.

### Theoretical Domains Framework (TDF) with other behavioural models and frameworks

Three studies combined TDF with the COM-B and Behaviour Change Wheel (BCW), with two focusing on PPE usage practice and one on multiple IPC practices. The TDF provides a detailed exploration of psychological and environmental factors influencing behaviour, aligning closely with the COM-B model’s components of Capability, Opportunity, and Motivation. The BCW utilises insights from both TDF and COM-B to guide the systematic development of interventions, mapping specific behaviour change techniques to the identified influences for effective and targeted strategies (Michie et al., 2011).

#### Personal protective equipment usage

Two studies combined TDF, COM-B, and BCW to identify the barriers related to PPE use and develop behaviour change interventions.

Curtis et al. (2022) identified 73 barriers to PPE use among HCWs, categorised into TDF domains like social influence, environmental context, resource availability, knowledge, emotion, memory, attention, and decision-making. These domains were then mapped to nine intervention functions using BCW and 42 behaviour change techniques (BCTs) to develop a strategy to improve PPE compliance. A PPE Marshal role was implemented as part of the behaviour change strategy, which observed staff and intervened when needed. The PPE Marshal further identified the most common reasons for noncompliance with PPE regulations, such as a lack of access to a buddy, depleted PPE stock, improper attire, and incorrect use of PPE.

Likewise, Castro-Sánchez et al. (2021) used a TDF and COM-B model–based survey to explore how a PPE intervention (the PPE Helper program) was impacting HCWs. The PPE Helper program aimed to enhance PPE use, addressing challenges identified through a previous study which used the COM-B model. This survey which used hospital staff as participants found that supply, inconsistent advice, training issues, and distrust in guidance were barriers to HCWs’ compliance with PPE. However, staff exposed to a PPE helper reported more positive knowledge and attitudes towards PPE, including confidence in the use of PPE, satisfaction with the availability and visibility of PPE in clinical areas, and less anxiety around PPE.

These two studies support how a theory-based IPC intervention can be rapidly developed and implemented to improve PPE practice among HCWs in hospitals during a pandemic.

#### Multiple IPC practices

Although no interventions were developed, Müller et al. (2021) also used TDF and COM-B-based interview guides to explore IPC practices among dentists and identified 34 enablers and 20 barriers to implementing IPC practices. Key barriers included a lack of knowledge, a lack of guidelines, and PPE recommendations, as well as limited availability of PPE and high PPE costs. Staff and patient pressure to prioritise infection control acted as enablers.

### Protection Motivation Theory (PMT)

The PMT (Rogers, 1975) explains how behavioural change occurs through cognitive processes involving threat and coping appraisal. Threat appraisal assesses the perceived severity of a disease and one’s vulnerability, while coping appraisal centres on the belief that recommended measures can mitigate the threat (response efficacy) and confidence in successfully implementing those actions (self-efficacy). Three studies used PMT to explore multiple IPC practices among HCWs.

#### Multiple IPC practices

Toghanian et al. (2022) explored COVID-19 preventive behaviours and their related factors using a PMT-based questionnaire. Findings revealed that protection motivation/intention and self-efficacy were significant predictors of IPC behaviours (*p* < .001). Likewise, in a similar study by Mortada et al. (2021), findings revealed significant positive correlations between COVID-19 behavioural intention and PMT constructs, with self-efficacy as the significant predictor of IPC behaviour (*p* = .008).

Bashirian et al. (2020) used PMT to predict the COVID-19 preventive behaviours of HCWs. Results showed that threat (perceived severity and vulnerability to COVID) and coping appraisal (belief in IPC measures and confidence in implementing them) predicted HCWs’ behavioural intention to engage in COVID-19 preventive behaviours (*p* < .001). All these three studies confirm the significance of the PMT model in predicting the IPC behaviours of HCWs.

The studies highlight the need to address threat perception, promote coping strategies, and enhance self-efficacy in interventions for promoting COVID-19 preventive behaviours among HCWs.

### Theory of Planned Behaviour (TPB)

The TPB (Ajzen, 1991) predicts behaviours based on intention (motivation for behaviour), attitude (favourable or unfavourable perception of the behaviour), subjective norm (social pressure to perform or avoid the behaviour), and perceived behavioural control (perception of ease or difficulty in carrying out the behaviour). Two studies employed the TPB to investigate different IPC practices of HCWs.

#### Hand hygiene

Sin and Rochelle (2022) used a TPB-based survey to investigate nurses’ hand hygiene behaviour. Results indicated that subjective norms (social pressure to perform or avoid hand hygiene) and perceived behavioural control (perception of ease or difficulty in performing hand hygiene) positively influenced hand hygiene behaviour through intentions.

#### Personal protective equipment usage

Seitz et al. (2021) assessed HCWs’ PPE practice using a TPB-based survey. Results showed high self-reported compliance with PPE use among HCWs, though some found PPE inconvenient (47.6%) or interfering with patient care (28.2%). Poor beliefs about PPE’s protective effects were observed among a third of HCWs. This study suggests that while HCWs report high compliance with PPE usage, there are concerns about PPE convenience and effectiveness.

### Health Action Process Approach (HAPA)

The HAPA model consists of a motivation phase, where individuals focus on developing the intention to engage in healthy behaviour, and the volition phase, where individuals move from having the intention to putting that intention into action (Schwarzer, 2008).

#### Hand hygiene

Derksen et al. (2020) used a HAPA model–based questionnaire to observe HCW adherence and assess determinants of hand hygiene behaviour. Findings showed that self-efficacy (confidence in practising better hand hygiene) was positively associated with the intention to wash or sanitise hands. Self-efficacy was the only determinant significantly associated with intention and hand hygiene behaviour.

### Quality of the included studies

The MMAT score for each included study is presented in the Supplemental File 6.

For quantitative studies (*n* = 14), overall, most followed the MMAT checklist criteria. However, methodological issues arose in two primary areas: appropriateness of measurements and bias considerations. Half of the studies used online surveys, which may not provide as representative results as face-to-face surveys (Szolnoki and Hoffmann, 2013). Additionally, social media platforms were used for participant recruitment, which can lead to selection bias (Regmi et al., 2017). Also, except for one study (Derksen et al., 2020), all others were based on self-reported IPC behaviours. Therefore, there is a high probability of recall bias, which can jeopardise the internal validity of the research (Hassan, 2006).

All three qualitative studies met the MMAT checklist criteria. However, only one specified the type of qualitative approach used. Among the two mixed-method studies, one met at least three MMAT checklist criteria, while the other only reported quantitative results, so only its quantitative part was assessed using the MMAT criteria.

## Discussion

This review examined the application of behaviour change theories, models, and frameworks to understand the factors influencing COVID-19 IPC practices of HCWs. Seven behaviour change theories, models, or frameworks were identified across the 19 included studies, with the Health Belief Model and Theoretical Domains Framework being the most adopted.

Health Belief Model (HBM)–based studies revealed cognitive factors associated with HCWs’ COVID-19 IPC practices. Emphasis on belief patterns like perceived severity and susceptibility, as demonstrated in studies by Jeong and Eun (2023), Kim and Kim (2023), Limkunakul et al. (2022), and Salwa et al. (2022), highlights the importance of addressing HCWs’ awareness of the risks associated with COVID-19 and the severity of its consequences. Perceived barriers to using PPE and self-efficacy (Salwa et al., 2022; Sivaraman et al., 2022) also emerge as crucial factors influencing IPC behaviours. These findings suggest that interventions to improve IPC practices should focus on increasing awareness of risks and addressing practical challenges that HCWs’ face. However, the HBM, while providing valuable insights across these studies, focuses primarily on individual beliefs and may not capture the broader organisational and social dynamics (Taylor et al., 2007) influencing HCWs’ IPC behaviours.

Theoretical Domains Framework (TDF)–based studies (Lohiniva et al., 2022; Silverberg et al., 2021; Van Hout et al., 2022; Yang et al., 2021) provided a nuanced understanding of the contextual, organisational, and cognitive factors influencing HCWs’ compliance with IPC practices. Studies by Castro-Sánchez et al. (2021) and Curtis et al. (2022) also combined TDF with the Behaviour Change Wheel (BCW) and COM-B model to develop interventions for improving PPE usage among HCWs. These studies highlight the importance of using frameworks like the TDF, COM-B, and BCW to develop comprehensive strategies that address cognitive and organisational factors to improve the IPC behaviours of HCWs. However, while the TDF helps identify potential factors influencing IPC behaviour, TDF, being a framework rather than a theory, does not propose testable relationships between elements and may not fully explain the behaviour change process (Atkins et al., 2017).

Studies based on the Protection Motivation Theory (PMT) (Bashirian et al., 2020; Mortada et al., 2021; Toghanian et al., 2022) shed light on the cognitive factors influencing HCWs’ motivation to engage in IPC behaviours. These studies suggest that PMT can effectively predict HCWs’ protection motivation and intention to adhere to IPC practices. The emphasis on threat and coping appraisals across these studies aligns with the idea that HCWs are more likely to adopt IPC measures when they perceive the severity and vulnerability of the threat and feel confident in their ability to cope. Thus, recognising HCWs’ confidence and belief in the effectiveness of IPC measures is crucial in designing interventions to improve compliance. However, like the HBM, PMT emphasises on individual cognition and may not sufficiently account for the influence of social and cultural factors (Plotnikoff and Trinh, 2010) on IPC behaviours of HCWs.

Studies based on the Theory of Planned Behaviour (TPB) (Seitz et al., 2021; Sin and Rochelle, 2022) highlighted the importance of intentions, attitudes, and perceived behavioural control in shaping HCWs’ IPC behaviours. However, despite high self-reported compliance with IPC measures across these studies, practical concerns like inconvenience related to PPE usage and hand hygiene revealed an intention–behaviour gap. Bridging this gap requires interventions that address these practical concerns and provide education and support to ensure proper IPC practices. It is important to note that while TPB has been useful in predicting IPC behaviours, it does not fully consider external factors like environmental constraints and social context (Taylor et al., 2007) that can also influence IPC behaviour.

A Health Action Process Approach (HAPA) model–based study (Derksen et al., 2020) found that building self-efficacy is crucial to enhancing the likelihood of HCWs translating their intentions into actual hand hygiene behaviours. However, it is essential to note that emotional factors, such as fear, stress, or anxiety, can significantly impact HCWs’ behaviours, especially in the context of a pandemic. This model does not explicitly address these emotional aspects (Schwarzer, 2016), potentially limiting its ability to explain and predict IPC behaviour in high-stress situations comprehensively.

Comparing behaviour change theories, models, and frameworks identified within this review, it becomes evident that each provides valuable insights into the factors influencing IPC practices of HCWs. The Health Belief Model, Protection Motivation Theory, and Theory of Planned Behaviour offer insights into individual perceptions and motivations behind HCWs’ IPC practices. The Health Action Process Approach explores social and cognitive influences, while the Theoretical Domains Framework and COM-B model comprehensively address cognitive, environmental, and social factors shaping IPC behaviours. Moreover, the Behaviour Change Wheel provides practical guidance for effective IPC interventions. Therefore, the choice among these theories in IPC research depends on research focus, whether on individual-level determinants or a holistic examination of IPC behaviour with attention to environmental and social influences (Weston et al., 2020). Integrating theories, models, and frameworks deepens the understanding of factors influencing IPC behaviours of HCWs during COVID-19 and beyond. Also, as seen in some studies, combining them can provide a foundation for targeted IPC interventions.

## Strengths and limitations

To the best of authors’ knowledge, this systematic review is the first comprehensive examination of behaviour change theories, models, or frameworks in COVID-19 research, focusing on HCWs’ IPC behaviour. However, it is essential to acknowledge certain limitations. Firstly, including only English-language studies may introduce reporting bias, potentially missing relevant research in other languages. Furthermore, although this review included studies from broad geographical locations, most were cross-sectional, which can establish connections but not causation (Wang and Cheng, 2020). Most studies also relied on self-reported data for HCWs’ IPC behaviours during COVID-19. However, during a pandemic, behaviours like hand hygiene can be sensitive, leading to social desirability bias, with respondents underreporting socially undesirable behaviours and overreporting desirable ones (Krumpal, 2013). Therefore, conclusions from these studies may be influenced by social desirability bias.

## Implications for future

This review highlights the importance of applying behaviour change theories to understand HCWs’ IPC behaviours. It identifies critical determinants like individual beliefs, organisational support, and environmental resources, emphasising areas that need attention in IPC practice implementation, especially during outbreaks. These determinants offer insights for policymakers and practitioners, guiding them towards effective IPC practices. The review also stresses the value of integrated theoretical frameworks like TDF and BCW in understanding and developing IPC interventions. The practical implications of these behaviour change theories in designing effective strategies to enhance IPC practices, which promote the safety of HCWs in ongoing and future public health crises, make continued research in this area crucial. Thus, future research should continue to explore the effectiveness of different behaviour change theories and their integration in developing tailored interventions to improve IPC practices among HCWs.

## Conclusion

This systematic review identified 19 studies using seven different behaviour change theories, models, and frameworks to understand the IPC practices of HCWs during COVID-19. These studies highlighted personal beliefs, environmental factors, and organisational support as crucial determinants of the IPC behaviour of HCWs. Addressing perceived susceptibility, severity, and self-efficacy was identified as critical across these studies. Furthermore, combining multiple frameworks across some studies provided a comprehensive understanding of the factors influencing the implementation of IPC practices among HCWs. IPC practitioners and researchers can benefit from using these theories to explore IPC practices and promote behavioural change.

## Supplemental Material


Supplemental Material - Theoretical models applied to understand infection prevention and control practices of healthcare workers during the COVID-19 pandemic: A systematic review
Supplemental Material for Theoretical models applied to understand infection prevention and control practices of healthcare workers during the COVID-19 pandemic: A systematic review by Deepti KC, Jan Smith, Kay Currie and Valerie Ness in Journal of Infection Prevention.

## Data Availability

All data analysed in this systematic review are included within this article, and also as supplementary files.*

## References

[bibr1-17571774241251645] Abed Alah M Abdeen S Selim N , et al. (2021) Compliance and barriers to the use of infection prevention and control measures among health care workers during COVID-19 pandemic in Qatar: a national survey. Journal of Nursing Management 29(8): 2401–2411. DOI: 10.1111/jonm.13440. 34351012 PMC8420516

[bibr2-17571774241251645] Ajzen I (1991) The theory of planned behavior. Organizational Behavior and Human Decision Processes 50(2): 179–211.

[bibr3-17571774241251645] Atkins L Francis J Islam R , et al. (2017) A guide to using the theoretical domains framework of behaviour change to investigate implementation problems. Implementation Science 12(1): 77. DOI: 10.1186/s13012-017-0605-9. 28637486 PMC5480145

[bibr5-17571774241251645] Bashirian S Jenabi E Khazaei S , et al. (2020) Factors associated with preventive behaviours of COVID-19 among hospital staff in Iran in 2020: an application of the protection motivation theory. Journal of Hospital Infection 105(3): 430–433. DOI: 10.1016/j.jhin.2020.04.035. 32360337 PMC7194681

[bibr6-17571774241251645] Cane J O’Connor D Michie S (2012) Validation of the theoretical domains framework for use in behaviour change and implementation research. Implementation Science 7(1): 1–17. DOI: 10.1186/1748-5908-7-37. PMC3483008 22530986

[bibr7-17571774241251645] Castro-Sánchez E CM A C A , et al. (2021) Evaluation of a personal protective equipment support programme for staff during the COVID-19 pandemic in London. Journal of Hospital Infection 109: 68–77. DOI: 10.1016/j.jhin.2020.12.004. 33307145 PMC7722521

[bibr8-17571774241251645] Curtis K Jansen P Mains M , et al. (2022) Rapid development and implementation of a behaviour change strategy to improve COVID-19 personal protective equipment use in a regional Australian emergency department. Australasian Emergency Care 25(4): 273–282. DOI: 10.1016/j.auec.2022.01.004. 35123929 PMC8802564

[bibr9-17571774241251645] Darnton A (2008) GSR behaviour change knowledge review reference report: an overview of behaviour change models and their uses. Available at: https://assets.publishing.service.gov.uk/government/uploads/system/uploads/attachment_data/file/498065/Behaviour_change_reference_report_tcm6-9697.pdf.

[bibr10-17571774241251645] Derksen C Keller FM Lippke S (2020) Obstetric healthcare workers’ adherence to hand hygiene recommendations during the COVID‐19 pandemic: observations and social-cognitive determinants. Applied Psychology: Health and Well-Being 12(4): 1286–1305. DOI: 10.1111/aphw.12240. 33016518 PMC7675238

[bibr11-17571774241251645] Greene C Wilson J (2022) The use of behaviour change theory for infection prevention and control practices in healthcare settings: a scoping review. Journal of Infection Prevention 23(3): 108–117. DOI: 10.1177/17571774211066779. 35495101 PMC9052851

[bibr12-17571774241251645] Hassan ES (2006) Recall bias can be a threat to retrospective and prospective research designs. The Internet Journal of Epidemiology 3(2). DOI: 10.5580/2732.

[bibr13-17571774241251645] Hong QN Gonzalez-Reyes A Pluye P (2018) Improving the usefulness of a tool for appraising the quality of qualitative, quantitative and mixed methods studies, the Mixed Methods Appraisal Tool (MMAT). Journal of Evaluation in Clinical Practice 24(3): 459–467. 29464873 10.1111/jep.12884

[bibr14-17571774241251645] Houghton C Meskell P Delaney H , et al. (2020) Barriers and facilitators to healthcare workers’ adherence with infection prevention and control (IPC) guidelines for respiratory infectious diseases: a rapid qualitative evidence synthesis. Cochrane Database of Systematic Reviews 4(4): CD013582. DOI: 10.1002/14651858.cd013582. 32315451 PMC7173761

[bibr15-17571774241251645] Jeong D Eun Y (2023) Factors influencing SARS-CoV-2 infection control practices of nurses caring for COVID-19 patients in South Korea: based on Health Belief Model. International Journal of Environmental Research and Public Health 20(4): 3223. DOI: 10.3390/ijerph20043223. 36833918 PMC9966129

[bibr16-17571774241251645] Kim SO Kim KH (2023) Factors influencing emergency nurses’ infection control practices related to coronavirus disease 2019 in Korea. Australasian Emergency Care 26(1): 30–35. DOI: 10.1016/j.auec.2022.07.004. 35872086 PMC9271496

[bibr17-17571774241251645] Krumpal I (2013) Determinants of social desirability bias in sensitive surveys: a literature review. Quality and Quantity: International Journal of Methodology 47(4): 2025–2047. Available at: https://ideas.repec.org/a/spr/qualqt/v47y2013i4p2025-2047.html (accessed 8 September 2022).

[bibr18-17571774241251645] Limkunakul C Phuthomdee S Srinithiwat P , et al. (2022) Factors associated with preventive behaviors for COVID-19 infection among healthcare workers by a health behaviour model. Tropical Medicine and Health 50(1): 1–7. DOI: 10.1186/s41182-022-00454-z. 36071539 PMC9449286

[bibr19-17571774241251645] Lohiniva A-L Toura S Arifulla D , et al. (2022) Exploring behavioural factors influencing COVID-19-specific infection prevention and control measures in Finland: a mixed-methods study, December 2020 to March 2021. Euro Surveillance 27(40): 2100915. DOI: 10.2807/1560-7917.es.2022.27.40.2100915. 36205170 PMC9540522

[bibr20-17571774241251645] Michie S Johnston M Abraham C , et al. (2005) Making psychological theory useful for implementing evidence based practice: a consensus approach. Quality and Safety in Health Care 14(1): 26–33. DOI: 10.1136/qshc.2004.011155. 15692000 PMC1743963

[bibr21-17571774241251645] Michie S van Stralen MM West R (2011) The behaviour change wheel: a new method for characterising and designing behaviour change interventions. Implementation Science 6(42): 1–11. DOI: 10.1186/1748-5908-6-42. 21513547 PMC3096582

[bibr22-17571774241251645] Michie S West R Campbell R , et al. (2014) ABC of Behaviour Change Theories: [an Essential Resource for Researchers, Policy Makers and Practitioners; 83 Theories]. London, UK. Silverback.

[bibr23-17571774241251645] Mortada E Abdel-Azeem A Al SA , et al. (2021) Preventive behaviors towards COVID-19 pandemic among healthcare providers in Saudi Arabia using the Protection Motivation Theory. Risk Management and Healthcare Policy 14: 685–694. DOI: 10.2147/rmhp.s289837. 33628067 PMC7898786

[bibr24-17571774241251645] Müller A Melzow FS Göstemeyer G , et al. (2021) Implementation of COVID-19 infection control measures by German dentists: a qualitative study to identify enablers and barriers. International Journal of Environmental Research and Public Health 18(11): 5710. DOI: 10.3390/ijerph18115710. 34073452 PMC8198934

[bibr25-17571774241251645] Nilsen P (2015) Making sense of implementation theories, models and frameworks. Implementation Science 10(1): 1–13. DOI: 10.1186/s13012-015-0242-0. 25895742 PMC4406164

[bibr26-17571774241251645] Paez A (2017) Gray literature: an important resource in systematic reviews. Journal of Evidence-Based Medicine 10(3): 233–240. DOI: 10.1111/jebm.12266. 28857505

[bibr27-17571774241251645] Page MJ McKenzie JE Bossuyt PM , et al. (2021) The PRISMA 2020 statement: an updated guideline for reporting systematic reviews. British Medical Journal 372(71): n71. DOI: 10.1136/bmj.n71. 33782057 PMC8005924

[bibr28-17571774241251645] Plotnikoff RC Trinh L (2010) Protection motivation theory. Exercise and Sport Sciences Reviews 38(2): 91–98. DOI: 10.1097/jes.0b013e3181d49612. 20335741

[bibr29-17571774241251645] Popay J Roberts H Sowden A , et al. (2006) Guidance on the conduct of narrative synthesis in systematic reviews: a product from the ESRC methods programme. Available at: https://www.lancaster.ac.uk/media/lancaster-university/content-assets/documents/fhm/dhr/chir/NSsynthesisguidanceVersion1-April2006.pdf.

[bibr30-17571774241251645] Regmi PR Waithaka E Paudyal A , et al. (2017) Guide to the design and application of online questionnaire surveys. Nepal Journal of Epidemiology 6(4): 640–644, Available at: https://www.ncbi.nlm.nih.gov/pmc/articles/PMC5506389/. 10.3126/nje.v6i4.17258 PMC5506389 28804676

[bibr31-17571774241251645] Rogers RW (1975) A protection motivation theory of fear appeals and attitude change. Journal of Psychology 91(1): 93–114. DOI: 10.1080/00223980.1975.9915803. 28136248

[bibr32-17571774241251645] Rosenstock IM (1974) The health belief model and preventive health behavior. Health Education Monographs 2(4): 354–386. DOI: 10.1177/109019817400200405. 299611

[bibr33-17571774241251645] Salwa M Haque MA Islam SS , et al. (2022) Compliance of healthcare workers with the infection prevention and control guidance in tertiary care hospitals: quantitative findings from an explanatory sequential mixed-methods study in Bangladesh. BMJ Open 12(6): e054837. DOI: 10.1136/bmjopen-2021-054837. PMC9195156 35697439

[bibr34-17571774241251645] Schwarzer R (2008) Modeling Health Behavior Change: how to predict and modify the adoption and maintenance of health behaviors. Applied Psychology 57(1): 1–29. DOI: 10.1111/j.1464-0597.2007.00325.x.

[bibr35-17571774241251645] Schwarzer R (2016) Health Action Process Approach (HAPA) as a theoretical framework to understand behavior change. Actualidades en Psicología 30(121): 119–130, Available at: https://www.redalyc.org/journal/1332/133248870012/html/.

[bibr36-17571774241251645] Seitz RM Yaffee AQ Peacock E , et al. (2021) Self-reported use of personal protective equipment among emergency department nurses, physicians and advanced practice providers during the 2020 COVID-19 pandemic. International Journal of Environmental Research and Public Health 18(13): 7076. DOI: 10.3390/ijerph18137076. 34281013 PMC8297270

[bibr37-17571774241251645] Silverberg SL Puchalski Ritchie LM Gobat N , et al. (2021) COVID-19 infection prevention and control procedures and institutional trust: perceptions of Canadian intensive care and emergency department nurses. Canadian Journal of Anesthesia/Journal canadien d’anesthésie 68(8): 1165–1175. DOI: 10.1007/s12630-021-02028-9. PMC8158085 34046822

[bibr38-17571774241251645] Sin CS Rochelle TL (2022) Using the theory of planned behaviour to explain hand hygiene among nurses in Hong Kong during COVID-19. Journal of Hospital Infection 123: 119–125. DOI: 10.1016/j.jhin.2022.01.018. 35124145 PMC8812086

[bibr39-17571774241251645] Sivaraman G Lakshmanan J Paul B , et al. (2022) Shifting from anxiety to the new normal: a qualitative exploration on personal protective equipment use by otorhinolaryngology health-care professionals during COVID-19 pandemic. The Nigerian Postgraduate Medical Journal 29(2): 110–115. DOI: 10.4103/npmj.npmj_10_22. 35488578

[bibr40-17571774241251645] Smith LE Serfioti D Weston D , et al. (2021) Adherence to protective measures among healthcare workers in the UK: a cross-sectional study. Emergency Medicine Journal 39(2): 211454. DOI: 10.1136/emermed-2021-211454. PMC8788253 34848560

[bibr41-17571774241251645] Szolnoki G Hoffmann D (2013) Online, face-to-face and telephone surveys—comparing different sampling methods in wine consumer research. Wine Economics and Policy 2(2): 57–66. DOI: 10.1016/j.wep.2013.10.001.

[bibr42-17571774241251645] Taylor D Bury M Carter S , et al. (2007) A review of the use of the health belief model (HBM), the theory of reasoned action (TRA), the theory of planned behaviour (TPB) and the trans-theoretical model (TTM) to study and predict health related behaviour change. Available at: https://www.nice.org.uk/guidance/ph6/resources/behaviour-change-taylor-et-al-models-review2 (accessed 1 December 2022).

[bibr43-17571774241251645] Toghanian R Ghasemi S Hosseini M , et al. (2022) Protection behaviors and related factors against COVID-19 in the healthcare workers of the hospitals in Iran: a cross-sectional study. Iranian Journal of Nursing and Midwifery Research 27(6): 587–592. DOI: 10.4103/ijnmr.ijnmr_430_21. 36712308 PMC9881561

[bibr44-17571774241251645] Türktemiz H Ünal Ö Aydın DB (2021) Assessment of healthcare professionals’ perceptions and attitudes towards the COVID-19 pandemic in Turkey. Work 69(4): 1163–1170. DOI: 10.3233/wor-205305. 34420998

[bibr45-17571774241251645] van Hout D Hutchinson P Wanat M , et al. (2022) The experience of European hospital-based health care workers on following infection prevention and control procedures and their wellbeing during the first wave of the COVID-19 pandemic. PLoS One 17(2): e0245182. DOI: 10.1371/journal.pone.0245182. 35130294 PMC8820620

[bibr46-17571774241251645] Wang X Cheng Z (2020) Cross-sectional studies: strengths, weaknesses, and recommendations. Chest 158(1): 65–71. DOI: 10.1016/j.chest.2020.03.012. 32658654

[bibr47-17571774241251645] West R Michie S Rubin GJ , et al. (2020) Applying principles of behaviour change to reduce SARS-CoV-2 transmission. Nature Human Behaviour 4: 451–459. DOI: 10.1038/s41562-020-0887-9. 32377018

[bibr48-17571774241251645] Weston D Ip A Amlôt R (2020) Examining the application of behaviour change theories in the context of infectious disease outbreaks and emergency response: a review of reviews. BMC Public Health 20(1): 1–19. DOI: 10.1186/s12889-020-09519-2. 33004011 PMC7528712

[bibr49-17571774241251645] World Health Organization (2021) Infection prevention and control during health care when coronavirus disease (COVID-19) is suspected or confirmed. Available at: https://www.who.int/publications/i/item/WHO-2019-nCoV-IPC-2021.1 (accessed 12 November 2022).

[bibr50-17571774241251645] Yang Q Wang X Zhou Q , et al. (2021) Healthcare workers’ behaviors on infection prevention and control and their determinants during the COVID-19 pandemic: a cross-sectional study based on the Theoretical Domains Framework in Wuhan, China. Archives of Public Health 79(1): 1–10. DOI: 10.1186/s13690-021-00641-0. 34193306 PMC8242273

