# Supplementary material for: Theoretical models applied to understand infection prevention and control practices of healthcare workers during the COVID-19 pandemic: A systematic review
Source: J Infect Prev. 2024 May 16;26(1):33–43. doi: 10.1177/17571774241251645 (PMC11558798; doi:10.1177/17571774241251645)
Supplement: Supplemental Material - Theoretical models applied to understand infection prevention and control practices of healthcare workers during the COVID-19 pandemic: A systematic review [file sj-pdf-1-bji-10.1177_17571774241251645.pdf]

Supplementary File 1: PRISMA 2020 Checklist

Section and Topic	Item #	Checklist item	Location where item is reported
**TITLE**			
Title	1	Identify the report as a systematic review.	Page 1
**ABSTRACT**			
Abstract	2	See the PRISMA 2020 for Abstracts checklist.	Page 2
**INTRODUCTION**			
Rationale	3	Describe the rationale for the review in the context of existing knowledge.	Page 3-4
Objectives	4	Provide an explicit statement of the objective(s) or question(s) the review addresses.	Page 4
Eligibility criteria	5	Specify the inclusion and exclusion criteria for the review and how studies were grouped for the syntheses.	Page 4
Information sources	6	Specify all databases, registers, websites, organisations, reference lists and other sources searched or consulted to identify studies. Specify the date when each source was last searched or consulted.	Page 4-5
Search strategy	7	Present the full search strategies for all databases, registers and websites, including any filters and limits used.	Supplementary File 2
Selection process	8	Specify the methods used to decide whether a study met the inclusion criteria of the review, including how many reviewers screened each record and each report retrieved, whether they worked independently, and if applicable, details of automation tools used in the process.	Page 5
Data collection process	9	Specify the methods used to collect data from reports, including how many reviewers collected data from each report, whether they worked independently, any processes for obtaining or confirming data from study investigators, and if applicable, details of automation tools used in the process.	Page 5
Data items	10a	List and define all outcomes for which data were sought. Specify whether all results that were compatible with each outcome domain in each study were sought (e.g. for all measures, time points, analyses), and if not, the methods used to decide which results to collect.	Page 5
	10b	List and define all other variables for which data were sought (e.g. participant and intervention characteristics, funding sources). Describe any assumptions made about any missing or unclear information.	NA

Section and Topic	Item #	Checklist item	Location where item is reported
Study risk of bias assessment	11	Specify the methods used to assess risk of bias in the included studies, including details of the tool(s) used, how many reviewers assessed each study and whether they worked independently, and if applicable, details of automation tools used in the process.	Page 5
Effect measures	12	Specify for each outcome the effect measure(s) (e.g. risk ratio, mean difference) used in the synthesis or presentation of results.	NA
Synthesis methods	13a	Describe the processes used to decide which studies were eligible for each synthesis (e.g. tabulating the study intervention characteristics and comparing against the planned groups for each synthesis (item #5)).	NA
	13b	Describe any methods required to prepare the data for presentation or synthesis, such as handling of missing summary statistics, or data conversions.	NA
	13c	Describe any methods used to tabulate or visually display results of individual studies and syntheses.	NA
	13d	Describe any methods used to synthesize results and provide a rationale for the choice(s). If meta-analysis was performed, describe the model(s), method(s) to identify the presence and extent of statistical heterogeneity, and software package(s) used.	NA
	13e	Describe any methods used to explore possible causes of heterogeneity among study results (e.g. subgroup analysis, meta-regression).	NA
	13f	Describe any sensitivity analyses conducted to assess robustness of the synthesized results.	NA
Reporting bias assessment	14	Describe any methods used to assess risk of bias due to missing results in a synthesis (arising from reporting biases).	Page 5
Certainty assessment	15	Describe any methods used to assess certainty (or confidence) in the body of evidence for an outcome.	Page 5
**RESULTS**			
Study selection	16a	Describe the results of the search and selection process, from the number of records identified in the search to the number of studies included in the review, ideally using a flow diagram.	Page 6
	16b	Cite studies that might appear to meet the inclusion criteria, but which were excluded, and explain why they were excluded.	Supplementary File 3
Study characteristics	17	Cite each included study and present its characteristics.	Table 1
Risk of bias in	18	Present assessments of risk of bias for each included study.	Supplementary

Section and Topic	Item #	Checklist item	Location where item is reported
studies			File 6
Results of individual studies	19	For all outcomes, present, for each study: (a) summary statistics for each group (where appropriate) and (b) an effect estimate and its precision (e.g. confidence/credible interval), ideally using structured tables or plots.	Page 7-11
Results of syntheses	20a	For each synthesis, briefly summarise the characteristics and risk of bias among contributing studies.	NA
	20b	Present results of all statistical syntheses conducted. If meta-analysis was done, present for each the summary estimate and its precision (e.g. confidence/credible interval) and measures of statistical heterogeneity. If comparing groups, describe the direction of the effect.	NA
	20c	Present results of all investigations of possible causes of heterogeneity among study results.	NA
	20d	Present results of all sensitivity analyses conducted to assess the robustness of the synthesized results.	NA
Reporting biases	21	Present assessments of risk of bias due to missing results (arising from reporting biases) for each synthesis assessed.	NA
Certainty of evidence	22	Present assessments of certainty (or confidence) in the body of evidence for each outcome assessed.	NA
**DISCUSSION**			
Discussion	23a	Provide a general interpretation of the results in the context of other evidence.	Page 12-14
	23b	Discuss any limitations of the evidence included in the review.	Page 15
	23c	Discuss any limitations of the review processes used.	Page 15
	23d	Discuss implications of the results for practice, policy, and future research.	Page 16
**OTHER INFORMATION**			
Registration and protocol	24a	Provide registration information for the review, including register name and registration number, or state that the review was not registered.	Page 4
	24b	Indicate where the review protocol can be accessed, or state that a protocol was not prepared.	Page 4
	24c	Describe and explain any amendments to information provided at registration or in the protocol.	NA
Support	25	Describe sources of financial or non-financial support for the review, and the role of the funders or sponsors in the review.	Page 1
Competing interests	26	Declare any competing interests of review authors.	Page 16

Section and Topic	Item #	Checklist item	Location where item is reported
Availability of data, code and other materials	27	Report which of the following are publicly available and where they can be found: template data collection forms; data extracted from included studies; data used for all analyses; analytic code; any other materials used in the review.	Supplementary File 2-6

From: Page MJ, McKenzie JE, Bossuyt PM, Boutron I, Hoffmann TC, Mulrow CD, et al. The PRISMA 2020 statement: an updated guideline for reporting systematic reviews. BMJ 2021;372:n71. doi: 10.1136/bmj.n71

For more information, visit: http://www.prisma-statement.org/

Supplementary File 2: Full Search Strategy

**Search History (EBSCO HOST Interface)**				
	Friday, March 24, 2023 2:35:02 PM			
**#**	**Query**	**Limiters/Expanders**	**Last Run Via**	**Results**
S37	S2 AND S4 AND S27	Expanders - Apply equivalent subjects	Interface - EBSCOhost Research Databases	35
		Search modes - Find all my search terms	Search Screen - Advanced Search	
			Database - AMED - The Allied and Complementary Medicine Database;British Education Index;Child Development & Adolescent Studies;eBook Collection (EBSCOhost);Education Abstracts (H.W. Wilson);Educational Administration Abstracts;ERIC;GreenFILE;Health Source: Nursing/Academic Edition;Library, Information Science & Technology Abstracts;MEDLINE;Regional Business News;CINAHL Plus with Full Text;Business Source Premier;SPORTDiscus with Full Text	
S36	S2 AND S4 AND S25	Expanders - Apply equivalent subjects	Interface - EBSCOhost Research Databases	22

		Search modes - Find all my search terms	Search Screen - Advanced Search	
			Database - AMED - The Allied and Complementary Medicine Database;British Education Index;Child Development & Adolescent Studies;eBook Collection (EBSCOhost);Education Abstracts (H.W. Wilson);Educational Administration Abstracts;ERIC;GreenFILE;Health Source: Nursing/Academic Edition;Library, Information Science & Technology Abstracts;MEDLINE;Regional Business News;CINAHL Plus with Full Text;Business Source Premier;SPORTDiscus with Full Text	
S35	S2 AND S4 AND S22	Expanders - Apply equivalent subjects	Interface - EBSCOhost Research Databases	4
		Search modes - Find all my search terms	Search Screen - Advanced Search	
			Database - AMED - The Allied and Complementary Medicine Database;British Education Index;Child Development & Adolescent Studies;eBook Collection (EBSCOhost);Education Abstracts (H.W. Wilson);Educational Administration Abstracts;ERIC;GreenFILE;Health Source: Nursing/Academic Edition;Library, Information Science & Technology Abstracts;MEDLINE;Regional Business News;CINAHL Plus with Full Text;Business Source Premier;SPORTDiscus with Full Text	
S34	S2 AND S4 AND S21	Expanders - Apply equivalent subjects	Interface - EBSCOhost Research Databases	20
		Search modes - Find all my search terms	Search Screen - Advanced Search	
			Database - AMED - The Allied and Complementary Medicine Database;British Education Index;Child Development & Adolescent Studies;eBook Collection (EBSCOhost);Education Abstracts (H.W. Wilson);Educational Administration Abstracts;ERIC;GreenFILE;Health Source: Nursing/Academic Edition;Library, Information Science & Technology Abstracts;MEDLINE;Regional Business News;CINAHL Plus with Full Text;Business Source Premier;SPORTDiscus with Full Text	
S33	S2 AND S4 AND S19	Expanders - Apply equivalent subjects	Interface - EBSCOhost Research Databases	9
		Search modes - Find all my search terms	Search Screen - Advanced Search	
			Database - AMED - The Allied and Complementary Medicine Database;British Education Index;Child Development & Adolescent Studies;eBook Collection (EBSCOhost);Education Abstracts (H.W. Wilson);Educational Administration Abstracts;ERIC;GreenFILE;Health Source: Nursing/Academic Edition;Library, Information Science & Technology Abstracts;MEDLINE;Regional Business News;CINAHL Plus with Full Text;Business Source Premier;SPORTDiscus with Full Text	
S32	S2 AND S4	Expanders - Apply	Interface - EBSCOhost Research Databases	23

	AND S17	equivalent subjects		
		Search modes - Find all my search terms	Search Screen - Advanced Search	
			Database - AMED - The Allied and Complementary Medicine Database;British Education Index;Child Development & Adolescent Studies;eBook Collection (EBSCOhost);Education Abstracts (H.W. Wilson);Educational Administration Abstracts;ERIC;GreenFILE;Health Source: Nursing/Academic Edition;Library, Information Science & Technology Abstracts;MEDLINE;Regional Business News;CINAHL Plus with Full Text;Business Source Premier;SPORTDiscus with Full Text	
S31	S2 AND S4 AND S15	Expanders - Apply equivalent subjects	Interface - EBSCOhost Research Databases	282
		Search modes - Find all my search terms	Search Screen - Advanced Search	
			Database - AMED - The Allied and Complementary Medicine Database;British Education Index;Child Development & Adolescent Studies;eBook Collection (EBSCOhost);Education Abstracts (H.W. Wilson);Educational Administration Abstracts;ERIC;GreenFILE;Health Source: Nursing/Academic Edition;Library, Information Science & Technology Abstracts;MEDLINE;Regional Business News;CINAHL Plus with Full Text;Business Source Premier;SPORTDiscus with Full Text	
S30	S2 AND S4 AND S13	Expanders - Apply equivalent subjects	Interface - EBSCOhost Research Databases	107
		Search modes - Find all my search terms	Search Screen - Advanced Search	
			Database - AMED - The Allied and Complementary Medicine Database;British Education Index;Child Development & Adolescent Studies;eBook Collection (EBSCOhost);Education Abstracts (H.W. Wilson);Educational Administration Abstracts;ERIC;GreenFILE;Health Source: Nursing/Academic Edition;Library, Information Science & Technology Abstracts;MEDLINE;Regional Business News;CINAHL Plus with Full Text;Business Source Premier;SPORTDiscus with Full Text	
S29	S2 AND S4 AND S11	Expanders - Apply equivalent subjects	Interface - EBSCOhost Research Databases	33
		Search modes - Find all my search terms	Search Screen - Advanced Search	
			Database - AMED - The Allied and Complementary Medicine Database;British Education Index;Child Development & Adolescent Studies;eBook Collection (EBSCOhost);Education Abstracts (H.W. Wilson);Educational Administration Abstracts;ERIC;GreenFILE;Health Source: Nursing/Academic Edition;Library, Information Science & Technology Abstracts;MEDLINE;Regional Business News;CINAHL Plus with Full Text;Business Source Premier;SPORTDiscus with Full Text	

S28	S2 AND S4 AND S9	Expanders - Apply equivalent subjects	Interface - EBSCOhost Research Databases	193
		Search modes - Find all my search terms	Search Screen - Advanced Search	
			Database - AMED - The Allied and Complementary Medicine Database;British Education Index;Child Development & Adolescent Studies;eBook Collection (EBSCOhost);Education Abstracts (H.W. Wilson);Educational Administration Abstracts;ERIC;GreenFILE;Health Source: Nursing/Academic Edition;Library, Information Science & Technology Abstracts;MEDLINE;Regional Business News;CINAHL Plus with Full Text;Business Source Premier;SPORTDiscus with Full Text	
S27	AB health action process approach or hapa	Limiters - Published Date: 20190101-20230331	Interface - EBSCOhost Research Databases	3,167
		Expanders - Apply equivalent subjects	Search Screen - Advanced Search	
		Search modes - Find all my search terms	Database - AMED - The Allied and Complementary Medicine Database;British Education Index;Child Development & Adolescent Studies;eBook Collection (EBSCOhost);Education Abstracts (H.W. Wilson);Educational Administration Abstracts;ERIC;GreenFILE;Health Source: Nursing/Academic Edition;Library, Information Science & Technology Abstracts;MEDLINE;Regional Business News;CINAHL Plus with Full Text;Business Source Premier;SPORTDiscus with Full Text	
S26	TI health action process approach or hapa	Limiters - Published Date: 20190101-20230331	Interface - EBSCOhost Research Databases	272
		Expanders - Apply equivalent subjects	Search Screen - Advanced Search	
		Search modes - Find all my search terms	Database - AMED - The Allied and Complementary Medicine Database;British Education Index;Child Development & Adolescent Studies;eBook Collection (EBSCOhost);Education Abstracts (H.W. Wilson);Educational Administration Abstracts;ERIC;GreenFILE;Health Source: Nursing/Academic Edition;Library, Information Science & Technology Abstracts;MEDLINE;Regional Business News;CINAHL Plus with Full Text;Business Source Premier;SPORTDiscus with Full Text	
S25	AB theoretical domains framework or TDF	Limiters - Published Date: 20190101-20230331	Interface - EBSCOhost Research Databases	5,418
		Expanders - Apply equivalent subjects	Search Screen - Advanced Search	

		Search modes - Find all my search terms	Database - AMED - The Allied and Complementary Medicine Database;British Education Index;Child Development & Adolescent Studies;eBook Collection (EBSCOhost);Education Abstracts (H.W. Wilson);Educational Administration Abstracts;ERIC;GreenFILE;Health Source: Nursing/Academic Edition;Library, Information Science & Technology Abstracts;MEDLINE;Regional Business News;CINAHL Plus with Full Text;Business Source Premier;SPORTDiscus with Full Text	
S24	TI theoretical domains framework or TDF	Limiters - Published Date: 20190101-20230331	Interface - EBSCOhost Research Databases	3,053
		Expanders - Apply equivalent subjects	Search Screen - Advanced Search	
		Search modes - Find all my search terms	Database - AMED - The Allied and Complementary Medicine Database;British Education Index;Child Development & Adolescent Studies;eBook Collection (EBSCOhost);Education Abstracts (H.W. Wilson);Educational Administration Abstracts;ERIC;GreenFILE;Health Source: Nursing/Academic Edition;Library, Information Science & Technology Abstracts;MEDLINE;Regional Business News;CINAHL Plus with Full Text;Business Source Premier;SPORTDiscus with Full Text	
S23	TI behaviour change wheel or behavior change wheel BCW	Limiters - Published Date: 20190101-20230331	Interface - EBSCOhost Research Databases	251
		Expanders - Apply equivalent subjects	Search Screen - Advanced Search	
		Search modes - Find all my search terms	Database - AMED - The Allied and Complementary Medicine Database;British Education Index;Child Development & Adolescent Studies;eBook Collection (EBSCOhost);Education Abstracts (H.W. Wilson);Educational Administration Abstracts;ERIC;GreenFILE;Health Source: Nursing/Academic Edition;Library, Information Science & Technology Abstracts;MEDLINE;Regional Business News;CINAHL Plus with Full Text;Business Source Premier;SPORTDiscus with Full Text	
S22	AB behaviour change wheel or behavior change wheel BCW	Limiters - Published Date: 20190101-20230331	Interface - EBSCOhost Research Databases	675
		Expanders - Apply equivalent subjects	Search Screen - Advanced Search	
		Search modes - Find all my search terms	Database - AMED - The Allied and Complementary Medicine Database;British Education Index;Child Development & Adolescent Studies;eBook Collection (EBSCOhost);Education Abstracts (H.W. Wilson);Educational Administration Abstracts;ERIC;GreenFILE;Health Source: Nursing/Academic Edition;Library, Information Science & Technology Abstracts;MEDLINE;Regional Business News;CINAHL Plus with Full Text;Business Source Premier;SPORTDiscus with Full Text	

S21	AB com-b model or com-b or com b or com-b framework	Limiters - Published Date: 20190101-20230331	Interface - EBSCOhost Research Databases	2,532
		Expanders - Apply equivalent subjects	Search Screen - Advanced Search	
		Search modes - Find all my search terms	Database - AMED - The Allied and Complementary Medicine Database;British Education Index;Child Development & Adolescent Studies;eBook Collection (EBSCOhost);Education Abstracts (H.W. Wilson);Educational Administration Abstracts;ERIC;GreenFILE;Health Source: Nursing/Academic Edition;Library, Information Science & Technology Abstracts;MEDLINE;Regional Business News;CINAHL Plus with Full Text;Business Source Premier;SPORTDiscus with Full Text	
S20	TI com-b model or com-b or com b or com-b framework	Limiters - Published Date: 20190101-20230331	Interface - EBSCOhost Research Databases	2,532
		Expanders - Apply equivalent subjects	Search Screen - Advanced Search	
		Search modes - Find all my search terms	Database - AMED - The Allied and Complementary Medicine Database;British Education Index;Child Development & Adolescent Studies;eBook Collection (EBSCOhost);Education Abstracts (H.W. Wilson);Educational Administration Abstracts;ERIC;GreenFILE;Health Source: Nursing/Academic Edition;Library, Information Science & Technology Abstracts;MEDLINE;Regional Business News;CINAHL Plus with Full Text;Business Source Premier;SPORTDiscus with Full Text	
S19	AB protection motivation theory or pmt	Limiters - Published Date: 20190101-20230331	Interface - EBSCOhost Research Databases	2,150
		Expanders - Apply equivalent subjects	Search Screen - Advanced Search	
		Search modes - Find all my search terms	Database - AMED - The Allied and Complementary Medicine Database;British Education Index;Child Development & Adolescent Studies;eBook Collection (EBSCOhost);Education Abstracts (H.W. Wilson);Educational Administration Abstracts;ERIC;GreenFILE;Health Source: Nursing/Academic Edition;Library, Information Science & Technology Abstracts;MEDLINE;Regional Business News;CINAHL Plus with Full Text;Business Source Premier;SPORTDiscus with Full Text	
S18	TI protection motivation theory or pmt	Limiters - Published Date: 20190101-20230331	Interface - EBSCOhost Research Databases	1,811
		Expanders - Apply equivalent subjects	Search Screen - Advanced Search	

		Search modes - Find all my search terms	Database - AMED - The Allied and Complementary Medicine Database;British Education Index;Child Development & Adolescent Studies;eBook Collection (EBSCOhost);Education Abstracts (H.W. Wilson);Educational Administration Abstracts;ERIC;GreenFILE;Health Source: Nursing/Academic Edition;Library, Information Science & Technology Abstracts;MEDLINE;Regional Business News;CINAHL Plus with Full Text;Business Source Premier;SPORTDiscus with Full Text	
S17	AB social cognitive theory or sct or social-cognitive theory or social cognitive learning theory	Limiters - Published Date: 20190101-20230331	Interface - EBSCOhost Research Databases	12,853
		Expanders - Apply equivalent subjects	Search Screen - Advanced Search	
		Search modes - Find all my search terms	Database - AMED - The Allied and Complementary Medicine Database;British Education Index;Child Development & Adolescent Studies;eBook Collection (EBSCOhost);Education Abstracts (H.W. Wilson);Educational Administration Abstracts;ERIC;GreenFILE;Health Source: Nursing/Academic Edition;Library, Information Science & Technology Abstracts;MEDLINE;Regional Business News;CINAHL Plus with Full Text;Business Source Premier;SPORTDiscus with Full Text	
S16	TI social cognitive theory or sct or social-cognitive theory or social cognitive learning theory	Limiters - Published Date: 20190101-20230331	Interface - EBSCOhost Research Databases	10,023
		Expanders - Apply equivalent subjects	Search Screen - Advanced Search	
		Search modes - Find all my search terms	Database - AMED - The Allied and Complementary Medicine Database;British Education Index;Child Development & Adolescent Studies;eBook Collection (EBSCOhost);Education Abstracts (H.W. Wilson);Educational Administration Abstracts;ERIC;GreenFILE;Health Source: Nursing/Academic Edition;Library, Information Science & Technology Abstracts;MEDLINE;Regional Business News;CINAHL Plus with Full Text;Business Source Premier;SPORTDiscus with Full Text	
S15	AB transtheoretical model or trans-theoretical model or stages of change	Limiters - Published Date: 20190101-20230331	Interface - EBSCOhost Research Databases	72,140
		Expanders - Apply equivalent subjects	Search Screen - Advanced Search	
		Search modes - Find all my search terms	Database - AMED - The Allied and Complementary Medicine Database;British Education Index;Child Development & Adolescent Studies;eBook Collection (EBSCOhost);Education Abstracts (H.W. Wilson);Educational Administration Abstracts;ERIC;GreenFILE;Health Source: Nursing/Academic Edition;Library, Information Science & Technology Abstracts;MEDLINE;Regional Business News;CINAHL Plus with Full Text;Business Source Premier;SPORTDiscus with Full Text	

S14	TI transtheoretical model or trans-theoretical model or stages of change	Limiters - Published Date: 20190101-20230331	Interface - EBSCOhost Research Databases	71,871
		Expanders - Apply equivalent subjects	Search Screen - Advanced Search	
		Search modes - Find all my search terms	Database - AMED - The Allied and Complementary Medicine Database;British Education Index;Child Development & Adolescent Studies;eBook Collection (EBSCOhost);Education Abstracts (H.W. Wilson);Educational Administration Abstracts;ERIC;GreenFILE;Health Source: Nursing/Academic Edition;Library, Information Science & Technology Abstracts;MEDLINE;Regional Business News;CINAHL Plus with Full Text;Business Source Premier;SPORTDiscus with Full Text	
S13	AB theory of reasoned action or TRA	Limiters - Published Date: 20190101-20230331	Interface - EBSCOhost Research Databases	12,111
		Expanders - Apply equivalent subjects	Search Screen - Advanced Search	
		Search modes - Find all my search terms	Database - AMED - The Allied and Complementary Medicine Database;British Education Index;Child Development & Adolescent Studies;eBook Collection (EBSCOhost);Education Abstracts (H.W. Wilson);Educational Administration Abstracts;ERIC;GreenFILE;Health Source: Nursing/Academic Edition;Library, Information Science & Technology Abstracts;MEDLINE;Regional Business News;CINAHL Plus with Full Text;Business Source Premier;SPORTDiscus with Full Text	
S12	TI theory of reasoned action or TRA	Limiters - Published Date: 20190101-20230331	Interface - EBSCOhost Research Databases	11,741
		Expanders - Apply equivalent subjects	Search Screen - Advanced Search	
		Search modes - Find all my search terms	Database - AMED - The Allied and Complementary Medicine Database;British Education Index;Child Development & Adolescent Studies;eBook Collection (EBSCOhost);Education Abstracts (H.W. Wilson);Educational Administration Abstracts;ERIC;GreenFILE;Health Source: Nursing/Academic Edition;Library, Information Science & Technology Abstracts;MEDLINE;Regional Business News;CINAHL Plus with Full Text;Business Source Premier;SPORTDiscus with Full Text	
S11	AB theory of planned behavior or theory of planned behaviour or tpb	Limiters - Published Date: 20190101-20230331	Interface - EBSCOhost Research Databases	6,798
		Expanders - Apply equivalent subjects	Search Screen - Advanced Search	

		Search modes - Find all my search terms	Database - AMED - The Allied and Complementary Medicine Database;British Education Index;Child Development & Adolescent Studies;eBook Collection (EBSCOhost);Education Abstracts (H.W. Wilson);Educational Administration Abstracts;ERIC;GreenFILE;Health Source: Nursing/Academic Edition;Library, Information Science & Technology Abstracts;MEDLINE;Regional Business News;CINAHL Plus with Full Text;Business Source Premier;SPORTDiscus with Full Text	
S10	TI theory of planned behavior or theory of planned behaviour or tpb	Limiters - Published Date: 20190101-20230331	Interface - EBSCOhost Research Databases	6,202
		Expanders - Apply equivalent subjects	Search Screen - Advanced Search	
		Search modes - Find all my search terms	Database - AMED - The Allied and Complementary Medicine Database;British Education Index;Child Development & Adolescent Studies;eBook Collection (EBSCOhost);Education Abstracts (H.W. Wilson);Educational Administration Abstracts;ERIC;GreenFILE;Health Source: Nursing/Academic Edition;Library, Information Science & Technology Abstracts;MEDLINE;Regional Business News;CINAHL Plus with Full Text;Business Source Premier;SPORTDiscus with Full Text	
S9	AB health belief model or health belief theory or hbm	Limiters - Published Date: 20190101-20230331	Interface - EBSCOhost Research Databases	10,800
		Expanders - Apply equivalent subjects	Search Screen - Advanced Search	
		Search modes - Find all my search terms	Database - AMED - The Allied and Complementary Medicine Database;British Education Index;Child Development & Adolescent Studies;eBook Collection (EBSCOhost);Education Abstracts (H.W. Wilson);Educational Administration Abstracts;ERIC;GreenFILE;Health Source: Nursing/Academic Edition;Library, Information Science & Technology Abstracts;MEDLINE;Regional Business News;CINAHL Plus with Full Text;Business Source Premier;SPORTDiscus with Full Text	
S8	TI health belief model or health belief theory or hbm	Limiters - Published Date: 20190101-20230331	Interface - EBSCOhost Research Databases	6,810
		Expanders - Apply equivalent subjects	Search Screen - Advanced Search	
		Search modes - Find all my search terms	Database - AMED - The Allied and Complementary Medicine Database;British Education Index;Child Development & Adolescent Studies;eBook Collection (EBSCOhost);Education Abstracts (H.W. Wilson);Educational Administration Abstracts;ERIC;GreenFILE;Health Source: Nursing/Academic Edition;Library, Information Science & Technology Abstracts;MEDLINE;Regional Business News;CINAHL Plus with Full Text;Business Source Premier;SPORTDiscus with Full Text	

S7	S2 AND S4 AND S6	Limiters - Published Date: 20190101-20230331	Interface - EBSCOhost Research Databases	187
		Expanders - Apply equivalent subjects	Search Screen - Advanced Search	
		Search modes - Find all my search terms	Database - AMED - The Allied and Complementary Medicine Database;British Education Index;Child Development & Adolescent Studies;eBook Collection (EBSCOhost);Education Abstracts (H.W. Wilson);Educational Administration Abstracts;ERIC;GreenFILE;Health Source: Nursing/Academic Edition;Library, Information Science & Technology Abstracts;MEDLINE;Regional Business News;CINAHL Plus with Full Text;Business Source Premier;SPORTDiscus with Full Text	
S6	AB behaviour theory or behavioural theory or theory of behaviour or behavioural change theory or theory of behaviour change	Limiters - Published Date: 20190101-20230331	Interface - EBSCOhost Research Databases	57,137
		Expanders - Apply equivalent subjects	Search Screen - Advanced Search	
		Search modes - Find all my search terms	Database - AMED - The Allied and Complementary Medicine Database;British Education Index;Child Development & Adolescent Studies;eBook Collection (EBSCOhost);Education Abstracts (H.W. Wilson);Educational Administration Abstracts;ERIC;GreenFILE;Health Source: Nursing/Academic Edition;Library, Information Science & Technology Abstracts;MEDLINE;Regional Business News;CINAHL Plus with Full Text;Business Source Premier;SPORTDiscus with Full Text	
S5	TI behaviour theory or behavioural theory or theory of behaviour or behavioural change theory or theory of behaviour change	Limiters - Published Date: 20190101-20230331	Interface - EBSCOhost Research Databases	57,137
		Expanders - Apply equivalent subjects	Search Screen - Advanced Search	
		Search modes - Find all my search terms	Database - AMED - The Allied and Complementary Medicine Database;British Education Index;Child Development & Adolescent Studies;eBook Collection (EBSCOhost);Education Abstracts (H.W. Wilson);Educational Administration Abstracts;ERIC;GreenFILE;Health Source: Nursing/Academic Edition;Library, Information Science & Technology Abstracts;MEDLINE;Regional Business News;CINAHL Plus with Full Text;Business Source Premier;SPORTDiscus with Full Text	
S4	AB covid-19 or coronavirus or 2019-ncov or sars-cov-2 or cov-19	Limiters - Published Date: 20190101-20230331	Interface - EBSCOhost Research Databases	1,097,533
		Expanders - Apply equivalent subjects	Search Screen - Advanced Search	

		Search modes - Find all my search terms	Database - AMED - The Allied and Complementary Medicine Database;British Education Index;Child Development & Adolescent Studies;eBook Collection (EBSCOhost);Education Abstracts (H.W. Wilson);Educational Administration Abstracts;ERIC;GreenFILE;Health Source: Nursing/Academic Edition;Library, Information Science & Technology Abstracts;MEDLINE;Regional Business News;CINAHL Plus with Full Text;Business Source Premier;SPORTDiscus with Full Text	
S3	TI covid-19 or coronavirus or 2019-ncov or sars-cov-2 or cov-19	Limiters - Published Date: 20190101-20230331	Interface - EBSCOhost Research Databases	1,097,533
		Expanders - Apply equivalent subjects	Search Screen - Advanced Search	
		Search modes - Find all my search terms	Database - AMED - The Allied and Complementary Medicine Database;British Education Index;Child Development & Adolescent Studies;eBook Collection (EBSCOhost);Education Abstracts (H.W. Wilson);Educational Administration Abstracts;ERIC;GreenFILE;Health Source: Nursing/Academic Edition;Library, Information Science & Technology Abstracts;MEDLINE;Regional Business News;CINAHL Plus with Full Text;Business Source Premier;SPORTDiscus with Full Text	
S2	AB healthcare workers or healthcare professional or healthcare provider or healthcare personnel or doctor or nurse	Limiters - Published Date: 20190101-20230331	Interface - EBSCOhost Research Databases	508,733
		Expanders - Apply equivalent subjects	Search Screen - Advanced Search	
		Search modes - Find all my search terms	Database - AMED - The Allied and Complementary Medicine Database;British Education Index;Child Development & Adolescent Studies;eBook Collection (EBSCOhost);Education Abstracts (H.W. Wilson);Educational Administration Abstracts;ERIC;GreenFILE;Health Source: Nursing/Academic Edition;Library, Information Science & Technology Abstracts;MEDLINE;Regional Business News;CINAHL Plus with Full Text;Business Source Premier;SPORTDiscus with Full Text	
S1	TI healthcare workers or healthcare professional or healthcare provider or healthcare personnel or doctor or nurse	Limiters - Published Date: 20190101-20230331	Interface - EBSCOhost Research Databases	498,552
		Expanders - Apply equivalent subjects	Search Screen - Advanced Search	
		Search modes - Find all my search terms	Database - AMED - The Allied and Complementary Medicine Database;British Education Index;Child Development & Adolescent Studies;eBook Collection (EBSCOhost);Education Abstracts (H.W. Wilson);Educational Administration Abstracts;ERIC;GreenFILE;Health Source: Nursing/Academic Edition;Library, Information Science & Technology Abstracts;MEDLINE;Regional Business News;CINAHL Plus with Full Text;Business Source Premier;SPORTDiscus with Full Text	

PubMed Search History					
Search number	Query	Sort By	Filters	Search Details	Results
24	#1 AND #2 AND #13		from 2019 - 2023	(("health personnel"[MeSH Terms] OR ("health"[All Fields] AND "personnel"[All Fields]) OR "health personnel"[All Fields] OR ("healthcare"[All Fields] AND "workers"[All Fields]) OR "healthcare workers"[All Fields] OR ("health personnel"[MeSH Terms] OR ("health"[All Fields] AND "personnel"[All Fields]) OR "health personnel"[All Fields] OR ("healthcare"[All Fields] AND "professional"[All Fields]) OR "healthcare professional"[All Fields]) OR ("health personnel"[MeSH Terms] OR ("health"[All Fields] AND "personnel"[All Fields]) OR "health personnel"[All Fields] OR ("healthcare"[All Fields] AND "provider"[All Fields]) OR "healthcare provider"[All Fields]) OR (("delivery of health care"[MeSH Terms] OR ("delivery"[All Fields] AND "health"[All Fields] AND "care"[All Fields]) OR "delivery of health care"[All Fields] OR "healthcare"[All Fields] OR "healthcare s"[All Fields] OR "healthcares"[All Fields]) AND ("occupational groups"[MeSH Terms] OR ("occupational"[All Fields] AND "groups"[All Fields]) OR "occupational groups"[All Fields] OR "personnel"[All Fields] OR "personnel s"[All Fields] OR "personnels"[All Fields])) OR ("doctor s"[All Fields] OR "doctoral"[All Fields] OR "doctorally"[All Fields] OR "doctorate"[All Fields] OR "doctorates"[All Fields] OR "doctoring"[All Fields] OR "physicians"[MeSH Terms] OR "physicians"[All Fields] OR "doctor"[All Fields] OR "doctors"[All Fields]) OR ("nurse s"[All Fields] OR "nurses"[MeSH Terms] OR "nurses"[All Fields] OR "nurse"[All Fields] OR "nurses s"[All Fields])) AND 2019/01/01:2023/12/31[Date - Publication] AND (("covid 19"[All Fields] OR "covid 19"[MeSH Terms] OR "covid 19 vaccines"[All Fields] OR "covid 19 vaccines"[MeSH Terms] OR "covid 19 serotherapy"[All Fields] OR "covid 19 nucleic acid testing"[All Fields] OR "covid 19 nucleic acid testing"[MeSH Terms] OR "covid 19 serological testing"[All Fields] OR "covid 19 serological testing"[MeSH Terms] OR "covid 19 testing"[All Fields] OR "covid 19 testing"[MeSH Terms] OR "sars cov 2"[All Fields] OR "sars cov 2"[MeSH Terms] OR "severe acute respiratory syndrome coronavirus 2"[All Fields] OR "ncov"[All Fields] OR "2019 ncov"[All Fields] OR (("coronavirus"[MeSH Terms] OR "coronavirus"[All Fields] OR "cov"[All Fields]) AND 2019/11/01:3000/12/31[Date - Publication]) OR ("coronavirus"[MeSH Terms] OR "coronavirus"[All Fields] OR "coronaviruses"[All Fields]) OR ("sars cov 2"[MeSH Terms] OR "sars cov 2"[All Fields] OR "2019 ncov"[All Fields]) OR ("sars cov 2"[MeSH Terms] OR "sars cov 2"[All Fields]) OR "cov-19"[All Fields]) AND 2019/01/01:2023/12/31[Date - Publication]) AND (((("healthaction"[Journal] OR ("health"[All Fields] AND "action"[All Fields]) OR "health action"[All Fields]) AND ("process"[All Fields] OR "processe"[All Fields] OR "processed"[All Fields] OR "processes"[All Fields] OR "processing"[All Fields] OR "processings"[All Fields]) AND ("approach"[All Fields] OR "approach s"[All Fields] OR "approachability"[All Fields] OR "approachable"[All Fields] OR "approache"[All Fields] OR "approached"[All Fields] OR "approaches"[All Fields] OR "approaching"[All Fields] OR "approachs"[All Fields])) OR "hapa"[All Fields]) AND 2019/01/01:2023/12/31[Date - Publication])) AND (2019:2023[pdat])	36
23	#1 AND #2 AND #12		from 2019 - 2023	(("health personnel"[MeSH Terms] OR ("health"[All Fields] AND "personnel"[All Fields]) OR "health personnel"[All Fields] OR ("healthcare"[All Fields] AND "workers"[All Fields]) OR "healthcare workers"[All Fields] OR ("health personnel"[MeSH Terms] OR ("health"[All Fields] AND "personnel"[All Fields]) OR "health personnel"[All Fields] OR ("healthcare"[All Fields] AND "professional"[All Fields]) OR "healthcare professional"[All Fields]) OR ("health personnel"[MeSH Terms] OR ("health"[All Fields] AND "personnel"[All Fields]) OR "health personnel"[All Fields] OR ("healthcare"[All Fields] AND "provider"[All Fields]) OR "healthcare provider"[All Fields]) OR (("delivery of health care"[MeSH Terms] OR ("delivery"[All Fields] AND "health"[All Fields] AND "care"[All Fields]) OR "delivery of health care"[All Fields] OR "healthcare"[All Fields] OR "healthcare s"[All Fields] OR "healthcares"[All Fields]) AND ("occupational groups"[MeSH Terms] OR ("occupational"[All Fields] AND "groups"[All Fields]) OR "occupational groups"[All Fields] OR "personnel"[All Fields] OR "personnel s"[All Fields] OR "personnels"[All Fields])) OR ("doctor s"[All Fields] OR "doctoral"[All Fields] OR "doctorally"[All Fields] OR "doctorate"[All Fields] OR "doctorates"[All Fields] OR "doctoring"[All Fields] OR "physicians"[MeSH Terms] OR "physicians"[All Fields] OR	22

			"doctor"[All Fields] OR "doctors"[All Fields]) OR ("nurse s"[All Fields] OR "nurses"[MeSH Terms] OR "nurses"[All Fields] OR "nurse"[All Fields] OR "nurses s"[All Fields])) AND 2019/01/01:2023/12/31[Date - Publication] AND (("covid 19"[All Fields] OR "covid 19"[MeSH Terms] OR "covid 19 vaccines"[All Fields] OR "covid 19 vaccines"[MeSH Terms] OR "covid 19 serotherapy"[All Fields] OR "covid 19 nucleic acid testing"[All Fields] OR "covid 19 nucleic acid testing"[MeSH Terms] OR "covid 19 serological testing"[All Fields] OR "covid 19 serological testing"[MeSH Terms] OR "covid 19 testing"[All Fields] OR "covid 19 testing"[MeSH Terms] OR "sars cov 2"[All Fields] OR "sars cov 2"[MeSH Terms] OR "severe acute respiratory syndrome coronavirus 2"[All Fields] OR "ncov"[All Fields] OR "2019 ncov"[All Fields] OR (("coronavirus"[MeSH Terms] OR "coronavirus"[All Fields] OR "cov"[All Fields]) AND 2019/11/01:3000/12/31[Date - Publication]) OR ("coronavirus"[MeSH Terms] OR "coronavirus"[All Fields] OR "coronaviruses"[All Fields]) OR ("sars cov 2"[MeSH Terms] OR "sars cov 2"[All Fields] OR "2019 ncov"[All Fields]) OR ("sars cov 2"[MeSH Terms] OR "sars cov 2"[All Fields] OR "sars cov 2"[All Fields]) OR "cov-19"[All Fields]) AND 2019/01/01:2023/12/31[Date - Publication]) AND (((("theoretic"[All Fields] OR "theoretical"[All Fields] OR "theoretically"[All Fields]) AND ("domain s"[All Fields] OR "domains"[All Fields] OR "protein domains"[MeSH Terms] OR ("protein"[All Fields] AND "domains"[All Fields]) OR "protein domains"[All Fields] OR "domain"[All Fields]) AND ("framework"[All Fields] OR "framework s"[All Fields] OR "frameworks"[All Fields])) OR "TDF"[All Fields]) AND 2019/01/01:2023/12/31[Date - Publication])) AND (2019:2023[pdat])	
22	#1 AND #2 AND #11	from 2019 - 2023	(("health personnel"[MeSH Terms] OR ("health"[All Fields] AND "personnel"[All Fields]) OR "health personnel"[All Fields] OR ("healthcare"[All Fields] AND "workers"[All Fields]) OR "healthcare workers"[All Fields] OR ("health personnel"[MeSH Terms] OR ("health"[All Fields] AND "personnel"[All Fields]) OR "health personnel"[All Fields] OR ("healthcare"[All Fields] AND "professional"[All Fields]) OR "healthcare professional"[All Fields]) OR ("health personnel"[MeSH Terms] OR ("health"[All Fields] AND "personnel"[All Fields]) OR "health personnel"[All Fields] OR ("healthcare"[All Fields] AND "provider"[All Fields]) OR "healthcare provider"[All Fields]) OR (("delivery of health care"[MeSH Terms] OR ("delivery"[All Fields] AND "health"[All Fields] AND "care"[All Fields]) OR "delivery of health care"[All Fields] OR "healthcare"[All Fields] OR "healthcare s"[All Fields] OR "healthcares"[All Fields]) AND ("occupational groups"[MeSH Terms] OR ("occupational"[All Fields] AND "groups"[All Fields]) OR "occupational groups"[All Fields] OR "personnel"[All Fields] OR "personnel s"[All Fields] OR "personnels"[All Fields])) OR ("doctor s"[All Fields] OR "doctoral"[All Fields] OR "doctorally"[All Fields] OR "doctorate"[All Fields] OR "doctorates"[All Fields] OR "doctoring"[All Fields] OR "physicians"[MeSH Terms] OR "physicians"[All Fields] OR "doctor"[All Fields] OR "doctors"[All Fields]) OR ("nurse s"[All Fields] OR "nurses"[MeSH Terms] OR "nurses"[All Fields] OR "nurse"[All Fields] OR "nurses s"[All Fields])) AND 2019/01/01:2023/12/31[Date - Publication] AND (("covid 19"[All Fields] OR "covid 19"[MeSH Terms] OR "covid 19 vaccines"[All Fields] OR "covid 19 vaccines"[MeSH Terms] OR "covid 19 serotherapy"[All Fields] OR "covid 19 nucleic acid testing"[All Fields] OR "covid 19 nucleic acid testing"[MeSH Terms] OR "covid 19 serological testing"[All Fields] OR "covid 19 serological testing"[MeSH Terms] OR "covid 19 testing"[All Fields] OR "covid 19 testing"[MeSH Terms] OR "sars cov 2"[All Fields] OR "sars cov 2"[MeSH Terms] OR "severe acute respiratory syndrome coronavirus 2"[All Fields] OR "ncov"[All Fields] OR "2019 ncov"[All Fields] OR (("coronavirus"[MeSH Terms] OR "coronavirus"[All Fields] OR "cov"[All Fields]) AND 2019/11/01:3000/12/31[Date - Publication]) OR ("coronavirus"[MeSH Terms] OR "coronavirus"[All Fields] OR "coronaviruses"[All Fields]) OR ("sars cov 2"[MeSH Terms] OR "sars cov 2"[All Fields] OR "2019 ncov"[All Fields]) OR ("sars cov 2"[MeSH Terms] OR "sars cov 2"[All Fields] OR "sars cov 2"[All Fields]) OR "cov-19"[All Fields]) AND 2019/01/01:2023/12/31[Date - Publication]) AND (((("behav change"[Journal] OR ("behaviour"[All Fields] AND "change"[All Fields]) OR "behaviour change"[All Fields]) AND ("wheel"[All Fields] OR "wheeled"[All Fields] OR "wheeling"[All Fields] OR "wheels"[All Fields])) OR (("behavior"[MeSH Terms] OR "behavior"[All Fields] OR "behavioral"[All Fields] OR "behavioural"[All Fields] OR "behavior s"[All Fields] OR "behaviorally"[All Fields] OR "behaviour"[All Fields] OR "behaviourally"[All Fields] OR "behaviours"[All Fields] OR "behaviors"[All Fields] OR "pattern"[All Fields] OR "pattern s"[All Fields] OR "patternability"[All Fields] OR "patternable"[All Fields] OR "patterned"[All Fields] OR "patterning"[All Fields] OR "patternings"[All Fields] OR "patterns"[All Fields]) AND ("change"[All Fields] OR "changed"[All Fields] OR "changes"[All Fields] OR "changing"[All Fields] OR "changings"[All Fields]) AND ("wheel"[All Fields] OR "wheeled"[All Fields] OR	4

			"wheeling"[All Fields] OR "wheels"[All Fields]) AND "BCW"[All Fields])) AND 2019/01/01:2023/12/31[Date - Publication])) AND (2019:2023[pdat])	
21	#1 AND #2 AND #10	from 2019 - 2023	(("health personnel"[MeSH Terms] OR ("health"[All Fields] AND "personnel"[All Fields]) OR "health personnel"[All Fields] OR ("healthcare"[All Fields] AND "workers"[All Fields]) OR "healthcare workers"[All Fields] OR ("health personnel"[MeSH Terms] OR ("health"[All Fields] AND "personnel"[All Fields]) OR "health personnel"[All Fields] OR ("healthcare"[All Fields] AND "professional"[All Fields]) OR "healthcare professional"[All Fields]) OR ("health personnel"[MeSH Terms] OR ("health"[All Fields] AND "personnel"[All Fields]) OR "health personnel"[All Fields] OR ("healthcare"[All Fields] AND "provider"[All Fields]) OR "healthcare provider"[All Fields]) OR (("delivery of health care"[MeSH Terms] OR ("delivery"[All Fields] AND "health"[All Fields] AND "care"[All Fields]) OR "delivery of health care"[All Fields] OR "healthcare"[All Fields] OR "healthcare s"[All Fields] OR "healthcares"[All Fields]) AND ("occupational groups"[MeSH Terms] OR ("occupational"[All Fields] AND "groups"[All Fields]) OR "occupational groups"[All Fields] OR "personnel"[All Fields] OR "personnel s"[All Fields] OR "personnels"[All Fields])) OR ("doctor s"[All Fields] OR "doctoral"[All Fields] OR "doctorally"[All Fields] OR "doctorate"[All Fields] OR "doctorates"[All Fields] OR "doctoring"[All Fields] OR "physicians"[MeSH Terms] OR "physicians"[All Fields] OR "doctor"[All Fields] OR "doctors"[All Fields]) OR ("nurse s"[All Fields] OR "nurses"[MeSH Terms] OR "nurses"[All Fields] OR "nurse"[All Fields] OR "nurses s"[All Fields])) AND 2019/01/01:2023/12/31[Date - Publication] AND (("covid 19"[All Fields] OR "covid 19"[MeSH Terms] OR "covid 19 vaccines"[All Fields] OR "covid 19 vaccines"[MeSH Terms] OR "covid 19 serotherapy"[All Fields] OR "covid 19 nucleic acid testing"[All Fields] OR "covid 19 nucleic acid testing"[MeSH Terms] OR "covid 19 serological testing"[All Fields] OR "covid 19 serological testing"[MeSH Terms] OR "covid 19 testing"[All Fields] OR "covid 19 testing"[MeSH Terms] OR "sars cov 2"[All Fields] OR "sars cov 2"[MeSH Terms] OR "severe acute respiratory syndrome coronavirus 2"[All Fields] OR "ncov"[All Fields] OR "2019 ncov"[All Fields] OR (("coronavirus"[MeSH Terms] OR "coronavirus"[All Fields] OR "cov"[All Fields]) AND 2019/11/01:3000/12/31[Date - Publication]) OR ("coronavirus"[MeSH Terms] OR "coronavirus"[All Fields] OR "coronaviruses"[All Fields]) OR ("sars cov 2"[MeSH Terms] OR "sars cov 2"[All Fields] OR "2019 ncov"[All Fields]) OR ("sars cov 2"[MeSH Terms] OR "sars cov 2"[All Fields] OR "sars cov 2"[All Fields]) OR "cov-19"[All Fields]) AND 2019/01/01:2023/12/31[Date - Publication]) AND ((("com-b"[All Fields] AND ("model"[All Fields] OR "model s"[All Fields] OR "modeled"[All Fields] OR "modeler"[All Fields] OR "modeler s"[All Fields] OR "modelers"[All Fields] OR "modeling"[All Fields] OR "modelings"[All Fields] OR "modelization"[All Fields] OR "modelizations"[All Fields] OR "modelize"[All Fields] OR "modelized"[All Fields] OR "modelled"[All Fields] OR "modeller"[All Fields] OR "modellers"[All Fields] OR "modelling"[All Fields] OR "modellings"[All Fields] OR "models"[All Fields])) OR "com-b"[All Fields] OR ("com"[All Fields] AND "b"[All Fields]) OR ("com-b"[All Fields] AND ("framework"[All Fields] OR "framework s"[All Fields] OR "frameworks"[All Fields]))) AND 2019/01/01:2023/12/31[Date - Publication])) AND (2019:2023[pdat])	181
20	#1 AND #2 AND #9	from 2019 - 2023	(("health personnel"[MeSH Terms] OR ("health"[All Fields] AND "personnel"[All Fields]) OR "health personnel"[All Fields] OR ("healthcare"[All Fields] AND "workers"[All Fields]) OR "healthcare workers"[All Fields] OR ("health personnel"[MeSH Terms] OR ("health"[All Fields] AND "personnel"[All Fields]) OR "health personnel"[All Fields] OR ("healthcare"[All Fields] AND "professional"[All Fields]) OR "healthcare professional"[All Fields]) OR ("health personnel"[MeSH Terms] OR ("health"[All Fields] AND "personnel"[All Fields]) OR "health personnel"[All Fields] OR ("healthcare"[All Fields] AND "provider"[All Fields]) OR "healthcare provider"[All Fields]) OR (("delivery of health care"[MeSH Terms] OR ("delivery"[All Fields] AND "health"[All Fields] AND "care"[All Fields]) OR "delivery of health care"[All Fields] OR "healthcare"[All Fields] OR "healthcare s"[All Fields] OR "healthcares"[All Fields]) AND ("occupational groups"[MeSH Terms] OR ("occupational"[All Fields] AND "groups"[All Fields]) OR "occupational groups"[All Fields] OR "personnel"[All Fields] OR "personnel s"[All Fields] OR "personnels"[All Fields])) OR ("doctor s"[All Fields] OR "doctoral"[All Fields] OR "doctorally"[All Fields] OR "doctorate"[All Fields] OR "doctorates"[All Fields] OR "doctoring"[All Fields] OR "physicians"[MeSH Terms] OR "physicians"[All Fields] OR "doctor"[All Fields] OR "doctors"[All Fields]) OR ("nurse s"[All Fields] OR "nurses"[MeSH Terms] OR "nurses"[All Fields] OR "nurse"[All Fields] OR "nurses s"[All Fields])) AND 2019/01/01:2023/12/31[Date - Publication] AND (("covid 19"[All Fields] OR "covid 19"[MeSH Terms] OR "covid 19 vaccines"[All Fields] OR "covid 19 vaccines"[MeSH Terms] OR "covid 19	20

			serotherapy"[All Fields] OR "covid 19 nucleic acid testing"[All Fields] OR "covid 19 nucleic acid testing"[MeSH Terms] OR "covid 19 serological testing"[All Fields] OR "covid 19 serological testing"[MeSH Terms] OR "covid 19 testing"[All Fields] OR "covid 19 testing"[MeSH Terms] OR "sars cov 2"[All Fields] OR "sars cov 2"[MeSH Terms] OR "severe acute respiratory syndrome coronavirus 2"[All Fields] OR "ncov"[All Fields] OR "2019 ncov"[All Fields] OR (("coronavirus"[MeSH Terms] OR "coronavirus"[All Fields] OR "cov"[All Fields]) AND 2019/11/01:3000/12/31[Date - Publication]) OR ("coronavirus"[MeSH Terms] OR "coronavirus"[All Fields] OR "coronaviruses"[All Fields]) OR ("sars cov 2"[MeSH Terms] OR "sars cov 2"[All Fields] OR "2019 ncov"[All Fields]) OR ("sars cov 2"[MeSH Terms] OR "sars cov 2"[All Fields] OR "sars cov 2"[All Fields]) OR "cov-19"[All Fields]) AND 2019/01/01:2023/12/31[Date - Publication]) AND (((("protect"[All Fields] OR "protected"[All Fields] OR "protecting"[All Fields] OR "protection"[All Fields] OR "protections"[All Fields] OR "protective agents"[Pharmacological Action] OR "protective agents"[MeSH Terms] OR ("protective"[All Fields] AND "agents"[All Fields]) OR "protective agents"[All Fields] OR "protectant"[All Fields] OR "protectants"[All Fields] OR "protective"[All Fields] OR "protectively"[All Fields] OR "protectiveness"[All Fields] OR "protectives"[All Fields] OR "protects"[All Fields]) AND ("motivate"[All Fields] OR "motivated"[All Fields] OR "motivates"[All Fields] OR "motivating"[All Fields] OR "motivation"[MeSH Terms] OR "motivation"[All Fields] OR "motivations"[All Fields] OR "motive"[All Fields] OR "motivational"[All Fields] OR "motivator"[All Fields] OR "motivators"[All Fields] OR "motives"[All Fields]) AND ("theorie"[All Fields] OR "theories"[All Fields] OR "theory"[All Fields] OR "theory s"[All Fields])) OR "pmt"[All Fields]) AND 2019/01/01:2023/12/31[Date - Publication])) AND (2019:2023[pdat])	
19	#1 AND #2 AND #8	from 2019 - 2023	(("health personnel"[MeSH Terms] OR ("health"[All Fields] AND "personnel"[All Fields]) OR "health personnel"[All Fields] OR ("healthcare"[All Fields] AND "workers"[All Fields]) OR "healthcare workers"[All Fields] OR ("health personnel"[MeSH Terms] OR ("health"[All Fields] AND "personnel"[All Fields]) OR "health personnel"[All Fields] OR ("healthcare"[All Fields] AND "professional"[All Fields]) OR "healthcare professional"[All Fields]) OR ("health personnel"[MeSH Terms] OR ("health"[All Fields] AND "personnel"[All Fields]) OR "health personnel"[All Fields] OR ("healthcare"[All Fields] AND "provider"[All Fields]) OR "healthcare provider"[All Fields]) OR (("delivery of health care"[MeSH Terms] OR ("delivery"[All Fields] AND "health"[All Fields] AND "care"[All Fields]) OR "delivery of health care"[All Fields] OR "healthcare"[All Fields] OR "healthcare s"[All Fields] OR "healthcares"[All Fields]) AND ("occupational groups"[MeSH Terms] OR ("occupational"[All Fields] AND "groups"[All Fields]) OR "occupational groups"[All Fields] OR "personnel"[All Fields] OR "personnel s"[All Fields] OR "personnels"[All Fields])) OR ("doctor s"[All Fields] OR "doctoral"[All Fields] OR "doctorally"[All Fields] OR "doctorate"[All Fields] OR "doctorates"[All Fields] OR "doctoring"[All Fields] OR "physicians"[MeSH Terms] OR "physicians"[All Fields] OR "doctor"[All Fields] OR "doctors"[All Fields]) OR ("nurse s"[All Fields] OR "nurses"[MeSH Terms] OR "nurses"[All Fields] OR "nurse"[All Fields] OR "nurses s"[All Fields])) AND 2019/01/01:2023/12/31[Date - Publication] AND (("covid 19"[All Fields] OR "covid 19"[MeSH Terms] OR "covid 19 vaccines"[All Fields] OR "covid 19 vaccines"[MeSH Terms] OR "covid 19 serotherapy"[All Fields] OR "covid 19 nucleic acid testing"[All Fields] OR "covid 19 nucleic acid testing"[MeSH Terms] OR "covid 19 serological testing"[All Fields] OR "covid 19 serological testing"[MeSH Terms] OR "covid 19 testing"[All Fields] OR "covid 19 testing"[MeSH Terms] OR "sars cov 2"[All Fields] OR "sars cov 2"[MeSH Terms] OR "severe acute respiratory syndrome coronavirus 2"[All Fields] OR "ncov"[All Fields] OR "2019 ncov"[All Fields] OR (("coronavirus"[MeSH Terms] OR "coronavirus"[All Fields] OR "cov"[All Fields]) AND 2019/11/01:3000/12/31[Date - Publication]) OR ("coronavirus"[MeSH Terms] OR "coronavirus"[All Fields] OR "coronaviruses"[All Fields]) OR ("sars cov 2"[MeSH Terms] OR "sars cov 2"[All Fields] OR "2019 ncov"[All Fields]) OR ("sars cov 2"[MeSH Terms] OR "sars cov 2"[All Fields] OR "sars cov 2"[All Fields]) OR "cov-19"[All Fields]) AND 2019/01/01:2023/12/31[Date - Publication]) AND (("psychological theory"[MeSH Terms] OR ("psychological"[All Fields] AND "theory"[All Fields]) OR "psychological theory"[All Fields] OR ("social"[All Fields] AND "cognitive"[All Fields] AND "theory"[All Fields]) OR "social cognitive theory"[All Fields] OR "sct"[All Fields] OR ("psychological theory"[MeSH Terms] OR ("psychological"[All Fields] AND "theory"[All Fields]) OR "psychological theory"[All Fields] OR ("social"[All Fields] AND "cognitive"[All Fields] AND "theory"[All Fields]) OR "social cognitive theory"[All Fields]) OR (("social behavior"[MeSH Terms] OR ("social"[All Fields] AND "behavior"[All Fields]) OR "social behavior"[All Fields] OR	159

			"sociality"[All Fields] OR "social"[All Fields] OR "socialisation"[All Fields] OR "socialization"[MeSH Terms] OR "socialization"[All Fields] OR "socialise"[All Fields] OR "socialised"[All Fields] OR "socialising"[All Fields] OR "socialities"[All Fields] OR "socializations"[All Fields] OR "socialize"[All Fields] OR "socialized"[All Fields] OR "socializers"[All Fields] OR "socializes"[All Fields] OR "socializing"[All Fields] OR "socially"[All Fields] OR "socials"[All Fields]) AND ("cognition"[MeSH Terms] OR "cognition"[All Fields] OR "cognitions"[All Fields] OR "cognitive"[All Fields] OR "cognitively"[All Fields] OR "cognitives"[All Fields]) AND ("learning"[MeSH Terms] OR "learning"[All Fields] OR "learn"[All Fields] OR "learned"[All Fields] OR "learning s"[All Fields] OR "learnings"[All Fields] OR "learns"[All Fields]) AND ("theorie"[All Fields] OR "theories"[All Fields] OR "theory"[All Fields] OR "theory s"[All Fields]))) AND 2019/01/01:2023/12/31[Date - Publication])) AND (2019:2023[pdat])	
18	#1 AND #2 AND #7	from 2019 - 2023	(("health personnel"[MeSH Terms] OR ("health"[All Fields] AND "personnel"[All Fields]) OR "health personnel"[All Fields] OR ("healthcare"[All Fields] AND "workers"[All Fields]) OR "healthcare workers"[All Fields] OR ("health personnel"[MeSH Terms] OR ("health"[All Fields] AND "personnel"[All Fields]) OR "health personnel"[All Fields] OR ("healthcare"[All Fields] AND "professional"[All Fields]) OR "healthcare professional"[All Fields]) OR ("health personnel"[MeSH Terms] OR ("health"[All Fields] AND "personnel"[All Fields]) OR "health personnel"[All Fields] OR ("healthcare"[All Fields] AND "provider"[All Fields]) OR "healthcare provider"[All Fields]) OR (("delivery of health care"[MeSH Terms] OR ("delivery"[All Fields] AND "health"[All Fields] AND "care"[All Fields]) OR "delivery of health care"[All Fields] OR "healthcare"[All Fields] OR "healthcare s"[All Fields] OR "healthcares"[All Fields]) AND ("occupational groups"[MeSH Terms] OR ("occupational"[All Fields] AND "groups"[All Fields]) OR "occupational groups"[All Fields] OR "personnel"[All Fields] OR "personnel s"[All Fields] OR "personnels"[All Fields])) OR ("doctor s"[All Fields] OR "doctoral"[All Fields] OR "doctorally"[All Fields] OR "doctorate"[All Fields] OR "doctorates"[All Fields] OR "doctoring"[All Fields] OR "physicians"[MeSH Terms] OR "physicians"[All Fields] OR "doctor"[All Fields] OR "doctors"[All Fields]) OR ("nurse s"[All Fields] OR "nurses"[MeSH Terms] OR "nurses"[All Fields] OR "nurse"[All Fields] OR "nurses s"[All Fields])) AND 2019/01/01:2023/12/31[Date - Publication] AND (("covid 19"[All Fields] OR "covid 19"[MeSH Terms] OR "covid 19 vaccines"[All Fields] OR "covid 19 vaccines"[MeSH Terms] OR "covid 19 serotherapy"[All Fields] OR "covid 19 nucleic acid testing"[All Fields] OR "covid 19 nucleic acid testing"[MeSH Terms] OR "covid 19 serological testing"[All Fields] OR "covid 19 serological testing"[MeSH Terms] OR "covid 19 testing"[All Fields] OR "covid 19 testing"[MeSH Terms] OR "sars cov 2"[All Fields] OR "sars cov 2"[MeSH Terms] OR "severe acute respiratory syndrome coronavirus 2"[All Fields] OR "ncov"[All Fields] OR "2019 ncov"[All Fields] OR (("coronavirus"[MeSH Terms] OR "coronavirus"[All Fields] OR "cov"[All Fields]) AND 2019/11/01:3000/12/31[Date - Publication]) OR ("coronavirus"[MeSH Terms] OR "coronavirus"[All Fields] OR "coronaviruses"[All Fields]) OR ("sars cov 2"[MeSH Terms] OR "sars cov 2"[All Fields] OR "2019 ncov"[All Fields]) OR ("sars cov 2"[MeSH Terms] OR "sars cov 2"[All Fields] OR "sars cov 2"[All Fields]) OR "cov-19"[All Fields]) AND 2019/01/01:2023/12/31[Date - Publication]) AND (("transtheoretical model"[MeSH Terms] OR ("transtheoretical"[All Fields] AND "model"[All Fields]) OR "transtheoretical model"[All Fields] OR ("trans-theoretical"[All Fields] AND ("model"[All Fields] OR "model s"[All Fields] OR "modeled"[All Fields] OR "modeler"[All Fields] OR "modeler s"[All Fields] OR "modelers"[All Fields] OR "modeling"[All Fields] OR "modelings"[All Fields] OR "modelization"[All Fields] OR "modelizations"[All Fields] OR "modelize"[All Fields] OR "modelized"[All Fields] OR "modelled"[All Fields] OR "modeller"[All Fields] OR "modellers"[All Fields] OR "modelling"[All Fields] OR "modellings"[All Fields] OR "models"[All Fields])) OR ("transtheoretical model"[MeSH Terms] OR ("transtheoretical"[All Fields] AND "model"[All Fields]) OR "transtheoretical model"[All Fields] OR ("stages"[All Fields] AND "change"[All Fields]) OR "stages of change"[All Fields])) AND 2019/01/01:2023/12/31[Date - Publication])) AND (2019:2023[pdat])	42
17	#1 AND #2 AND #6	from 2019 - 2023	(("health personnel"[MeSH Terms] OR ("health"[All Fields] AND "personnel"[All Fields]) OR "health personnel"[All Fields] OR ("healthcare"[All Fields] AND "workers"[All Fields]) OR "healthcare workers"[All Fields] OR ("health personnel"[MeSH Terms] OR ("health"[All Fields] AND "personnel"[All Fields]) OR "health personnel"[All Fields] OR ("healthcare"[All Fields] AND "professional"[All Fields]) OR "healthcare professional"[All Fields]) OR ("health personnel"[MeSH Terms] OR ("health"[All Fields] AND "personnel"[All Fields]) OR "health personnel"[All Fields] OR ("healthcare"[All Fields] AND "provider"[All	45

			Fields]) OR "healthcare provider"[All Fields]) OR (("delivery of health care"[MeSH Terms] OR ("delivery"[All Fields] AND "health"[All Fields] AND "care"[All Fields]) OR "delivery of health care"[All Fields] OR "healthcare"[All Fields] OR "healthcare s"[All Fields] OR "healthcares"[All Fields]) AND ("occupational groups"[MeSH Terms] OR ("occupational"[All Fields] AND "groups"[All Fields]) OR "occupational groups"[All Fields] OR "personnel"[All Fields] OR "personnel s"[All Fields] OR "personnels"[All Fields])) OR ("doctor s"[All Fields] OR "doctoral"[All Fields] OR "doctorally"[All Fields] OR "doctorate"[All Fields] OR "doctorates"[All Fields] OR "doctoring"[All Fields] OR "physicians"[MeSH Terms] OR "physicians"[All Fields] OR "doctor"[All Fields] OR "doctors"[All Fields]) OR ("nurse s"[All Fields] OR "nurses"[MeSH Terms] OR "nurses"[All Fields] OR "nurse"[All Fields] OR "nurses s"[All Fields])) AND 2019/01/01:2023/12/31[Date - Publication] AND (("covid 19"[All Fields] OR "covid 19"[MeSH Terms] OR "covid 19 vaccines"[All Fields] OR "covid 19 vaccines"[MeSH Terms] OR "covid 19 serotherapy"[All Fields] OR "covid 19 nucleic acid testing"[All Fields] OR "covid 19 nucleic acid testing"[MeSH Terms] OR "covid 19 serological testing"[All Fields] OR "covid 19 serological testing"[MeSH Terms] OR "covid 19 testing"[All Fields] OR "covid 19 testing"[MeSH Terms] OR "sars cov 2"[All Fields] OR "sars cov 2"[MeSH Terms] OR "severe acute respiratory syndrome coronavirus 2"[All Fields] OR "ncov"[All Fields] OR "2019 ncov"[All Fields] OR (("coronavirus"[MeSH Terms] OR "coronavirus"[All Fields] OR "cov"[All Fields]) AND 2019/11/01:3000/12/31[Date - Publication]) OR ("coronavirus"[MeSH Terms] OR "coronavirus"[All Fields] OR "coronaviruses"[All Fields]) OR ("sars cov 2"[MeSH Terms] OR "sars cov 2"[All Fields] OR "2019 ncov"[All Fields]) OR ("sars cov 2"[MeSH Terms] OR "sars cov 2"[All Fields] OR "sars cov 2"[All Fields]) OR "cov-19"[All Fields]) AND 2019/01/01:2023/12/31[Date - Publication]) AND (("theory of planned behavior"[MeSH Terms] OR ("theory"[All Fields] AND "planned"[All Fields] AND "behavior"[All Fields]) OR "theory of planned behavior"[All Fields] OR ("theory"[All Fields] AND "reasoned"[All Fields] AND "action"[All Fields]) OR "theory of reasoned action"[All Fields] OR "TRA"[All Fields]) AND 2019/01/01:2023/12/31[Date - Publication])) AND (2019:2023[pdat])	
16	#1 AND #2 AND #5	from 2019 - 2023	(("health personnel"[MeSH Terms] OR ("health"[All Fields] AND "personnel"[All Fields]) OR "health personnel"[All Fields] OR ("healthcare"[All Fields] AND "workers"[All Fields]) OR "healthcare workers"[All Fields] OR ("health personnel"[MeSH Terms] OR ("health"[All Fields] AND "personnel"[All Fields]) OR "health personnel"[All Fields] OR ("healthcare"[All Fields] AND "professional"[All Fields]) OR "healthcare professional"[All Fields]) OR ("health personnel"[MeSH Terms] OR ("health"[All Fields] AND "personnel"[All Fields]) OR "health personnel"[All Fields] OR ("healthcare"[All Fields] AND "provider"[All Fields]) OR "healthcare provider"[All Fields]) OR (("delivery of health care"[MeSH Terms] OR ("delivery"[All Fields] AND "health"[All Fields] AND "care"[All Fields]) OR "delivery of health care"[All Fields] OR "healthcare"[All Fields] OR "healthcare s"[All Fields] OR "healthcares"[All Fields]) AND ("occupational groups"[MeSH Terms] OR ("occupational"[All Fields] AND "groups"[All Fields]) OR "occupational groups"[All Fields] OR "personnel"[All Fields] OR "personnel s"[All Fields] OR "personnels"[All Fields])) OR ("doctor s"[All Fields] OR "doctoral"[All Fields] OR "doctorally"[All Fields] OR "doctorate"[All Fields] OR "doctorates"[All Fields] OR "doctoring"[All Fields] OR "physicians"[MeSH Terms] OR "physicians"[All Fields] OR "doctor"[All Fields] OR "doctors"[All Fields]) OR ("nurse s"[All Fields] OR "nurses"[MeSH Terms] OR "nurses"[All Fields] OR "nurse"[All Fields] OR "nurses s"[All Fields])) AND 2019/01/01:2023/12/31[Date - Publication] AND (("covid 19"[All Fields] OR "covid 19"[MeSH Terms] OR "covid 19 vaccines"[All Fields] OR "covid 19 vaccines"[MeSH Terms] OR "covid 19 serotherapy"[All Fields] OR "covid 19 nucleic acid testing"[All Fields] OR "covid 19 nucleic acid testing"[MeSH Terms] OR "covid 19 serological testing"[All Fields] OR "covid 19 serological testing"[MeSH Terms] OR "covid 19 testing"[All Fields] OR "covid 19 testing"[MeSH Terms] OR "sars cov 2"[All Fields] OR "sars cov 2"[MeSH Terms] OR "severe acute respiratory syndrome coronavirus 2"[All Fields] OR "ncov"[All Fields] OR "2019 ncov"[All Fields] OR (("coronavirus"[MeSH Terms] OR "coronavirus"[All Fields] OR "cov"[All Fields]) AND 2019/11/01:3000/12/31[Date - Publication]) OR ("coronavirus"[MeSH Terms] OR "coronavirus"[All Fields] OR "coronaviruses"[All Fields]) OR ("sars cov 2"[MeSH Terms] OR "sars cov 2"[All Fields] OR "2019 ncov"[All Fields]) OR ("sars cov 2"[MeSH Terms] OR "sars cov 2"[All Fields] OR "sars cov 2"[All Fields]) OR "cov-19"[All Fields]) AND 2019/01/01:2023/12/31[Date - Publication]) AND (("theory of planned behavior"[MeSH Terms] OR ("theory"[All Fields] AND "planned"[All Fields] AND "behavior"[All Fields]) OR "theory of planned behavior"[All Fields] OR ("theory of planned behavior"[MeSH Terms] OR ("theory"[All Fields] AND "planned"[All Fields] AND "behavior"[All Fields])	31

			OR "theory of planned behavior"[All Fields] OR ("theory"[All Fields] AND "planned"[All Fields] AND "behaviour"[All Fields]) OR "theory of planned behaviour"[All Fields]) OR "tpb"[All Fields]) AND 2019/01/01:2023/12/31[Date - Publication])) AND (2019:2023[pdat])	
15	#1 AND #2 AND #4	from 2019 - 2023	(("health personnel"[MeSH Terms] OR ("health"[All Fields] AND "personnel"[All Fields]) OR "health personnel"[All Fields] OR ("healthcare"[All Fields] AND "workers"[All Fields]) OR "healthcare workers"[All Fields] OR ("health personnel"[MeSH Terms] OR ("health"[All Fields] AND "personnel"[All Fields]) OR "health personnel"[All Fields] OR ("healthcare"[All Fields] AND "professional"[All Fields]) OR "healthcare professional"[All Fields]) OR ("health personnel"[MeSH Terms] OR ("health"[All Fields] AND "personnel"[All Fields]) OR "health personnel"[All Fields] OR ("healthcare"[All Fields] AND "provider"[All Fields]) OR "healthcare provider"[All Fields]) OR (("delivery of health care"[MeSH Terms] OR ("delivery"[All Fields] AND "health"[All Fields] AND "care"[All Fields]) OR "delivery of health care"[All Fields] OR "healthcare"[All Fields] OR "healthcare s"[All Fields] OR "healthcares"[All Fields]) AND ("occupational groups"[MeSH Terms] OR ("occupational"[All Fields] AND "groups"[All Fields]) OR "occupational groups"[All Fields] OR "personnel"[All Fields] OR "personnel s"[All Fields] OR "personnels"[All Fields])) OR ("doctor s"[All Fields] OR "doctoral"[All Fields] OR "doctorally"[All Fields] OR "doctorate"[All Fields] OR "doctorates"[All Fields] OR "doctoring"[All Fields] OR "physicians"[MeSH Terms] OR "physicians"[All Fields] OR "doctor"[All Fields] OR "doctors"[All Fields]) OR ("nurse s"[All Fields] OR "nurses"[MeSH Terms] OR "nurses"[All Fields] OR "nurse"[All Fields] OR "nurses s"[All Fields])) AND 2019/01/01:2023/12/31[Date - Publication] AND (("covid 19"[All Fields] OR "covid 19"[MeSH Terms] OR "covid 19 vaccines"[All Fields] OR "covid 19 vaccines"[MeSH Terms] OR "covid 19 serotherapy"[All Fields] OR "covid 19 nucleic acid testing"[All Fields] OR "covid 19 nucleic acid testing"[MeSH Terms] OR "covid 19 serological testing"[All Fields] OR "covid 19 serological testing"[MeSH Terms] OR "covid 19 testing"[All Fields] OR "covid 19 testing"[MeSH Terms] OR "sars cov 2"[All Fields] OR "sars cov 2"[MeSH Terms] OR "severe acute respiratory syndrome coronavirus 2"[All Fields] OR "ncov"[All Fields] OR "2019 ncov"[All Fields] OR (("coronavirus"[MeSH Terms] OR "coronavirus"[All Fields] OR "cov"[All Fields]) AND 2019/11/01:3000/12/31[Date - Publication]) OR ("coronavirus"[MeSH Terms] OR "coronavirus"[All Fields] OR "coronaviruses"[All Fields]) OR ("sars cov 2"[MeSH Terms] OR "sars cov 2"[All Fields] OR "2019 ncov"[All Fields]) OR ("sars cov 2"[MeSH Terms] OR "sars cov 2"[All Fields] OR "sars cov 2"[All Fields]) OR "cov-19"[All Fields]) AND 2019/01/01:2023/12/31[Date - Publication]) AND (("health belief model"[MeSH Terms] OR ("health"[All Fields] AND "belief"[All Fields] AND "model"[All Fields]) OR "health belief model"[All Fields] OR (("health"[MeSH Terms] OR "health"[All Fields] OR "health s"[All Fields] OR "healthful"[All Fields] OR "healthfulness"[All Fields] OR "healths"[All Fields]) AND ("belief s"[All Fields] OR "culture"[MeSH Terms] OR "culture"[All Fields] OR "belief"[All Fields] OR "beliefs"[All Fields]) AND ("theorie"[All Fields] OR "theories"[All Fields] OR "theory"[All Fields] OR "theory s"[All Fields])) OR "hbm"[All Fields]) AND 2019/01/01:2023/12/31[Date - Publication])) AND (2019:2023[pdat])	144
14	#1 AND #2 AND #3	from 2019 - 2023	(("health personnel"[MeSH Terms] OR ("health"[All Fields] AND "personnel"[All Fields]) OR "health personnel"[All Fields] OR ("healthcare"[All Fields] AND "workers"[All Fields]) OR "healthcare workers"[All Fields] OR ("health personnel"[MeSH Terms] OR ("health"[All Fields] AND "personnel"[All Fields]) OR "health personnel"[All Fields] OR ("healthcare"[All Fields] AND "professional"[All Fields]) OR "healthcare professional"[All Fields]) OR ("health personnel"[MeSH Terms] OR ("health"[All Fields] AND "personnel"[All Fields]) OR "health personnel"[All Fields] OR ("healthcare"[All Fields] AND "provider"[All Fields]) OR "healthcare provider"[All Fields]) OR (("delivery of health care"[MeSH Terms] OR ("delivery"[All Fields] AND "health"[All Fields] AND "care"[All Fields]) OR "delivery of health care"[All Fields] OR "healthcare"[All Fields] OR "healthcare s"[All Fields] OR "healthcares"[All Fields]) AND ("occupational groups"[MeSH Terms] OR ("occupational"[All Fields] AND "groups"[All Fields]) OR "occupational groups"[All Fields] OR "personnel"[All Fields] OR "personnel s"[All Fields] OR "personnels"[All Fields])) OR ("doctor s"[All Fields] OR "doctoral"[All Fields] OR "doctorally"[All Fields] OR "doctorate"[All Fields] OR "doctorates"[All Fields] OR "doctoring"[All Fields] OR "physicians"[MeSH Terms] OR "physicians"[All Fields] OR "doctor"[All Fields] OR "doctors"[All Fields]) OR ("nurse s"[All Fields] OR "nurses"[MeSH Terms] OR "nurses"[All Fields] OR "nurse"[All Fields] OR "nurses s"[All Fields])) AND 2019/01/01:2023/12/31[Date - Publication] AND (("covid 19"[All Fields] OR "covid 19"[MeSH Terms] OR "covid 19 vaccines"[All Fields] OR "covid 19 vaccines"[MeSH Terms] OR "covid 19	261

			serotherapy"[All Fields] OR "covid 19 nucleic acid testing"[All Fields] OR "covid 19 nucleic acid testing"[MeSH Terms] OR "covid 19 serological testing"[All Fields] OR "covid 19 serological testing"[MeSH Terms] OR "covid 19 testing"[All Fields] OR "covid 19 testing"[MeSH Terms] OR "sars cov 2"[All Fields] OR "sars cov 2"[MeSH Terms] OR "severe acute respiratory syndrome coronavirus 2"[All Fields] OR "ncov"[All Fields] OR "2019 ncov"[All Fields] OR (("coronavirus"[MeSH Terms] OR "coronavirus"[All Fields] OR "cov"[All Fields]) AND 2019/11/01:3000/12/31[Date - Publication]) OR ("coronavirus"[MeSH Terms] OR "coronavirus"[All Fields] OR "coronaviruses"[All Fields]) OR ("sars cov 2"[MeSH Terms] OR "sars cov 2"[All Fields] OR "2019 ncov"[All Fields]) OR ("sars cov 2"[MeSH Terms] OR "sars cov 2"[All Fields] OR "sars cov 2"[All Fields]) OR "cov-19"[All Fields]) AND 2019/01/01:2023/12/31[Date - Publication]) AND (((("behavior"[MeSH Terms] OR "behavior"[All Fields] OR "behavioral"[All Fields] OR "behavioural"[All Fields] OR "behavior s"[All Fields] OR "behaviorally"[All Fields] OR "behaviour"[All Fields] OR "behaviourally"[All Fields] OR "behaviours"[All Fields] OR "behaviors"[All Fields] OR "pattern"[All Fields] OR "pattern s"[All Fields] OR "patternability"[All Fields] OR "patternable"[All Fields] OR "patterned"[All Fields] OR "patterning"[All Fields] OR "patternings"[All Fields] OR "patterns"[All Fields]) AND ("theorie"[All Fields] OR "theories"[All Fields] OR "theory"[All Fields] OR "theory s"[All Fields])) OR (("behavior"[MeSH Terms] OR "behavior"[All Fields] OR "behavioral"[All Fields] OR "behavioural"[All Fields] OR "behavior s"[All Fields] OR "behaviorally"[All Fields] OR "behaviour"[All Fields] OR "behaviourally"[All Fields] OR "behaviours"[All Fields] OR "behaviors"[All Fields] OR "pattern"[All Fields] OR "pattern s"[All Fields] OR "patternability"[All Fields] OR "patternable"[All Fields] OR "patterned"[All Fields] OR "patterning"[All Fields] OR "patternings"[All Fields] OR "patterns"[All Fields]) AND ("theorie"[All Fields] OR "theories"[All Fields] OR "theory"[All Fields] OR "theory s"[All Fields])) OR (("behavior"[MeSH Terms] OR "behavior"[All Fields] OR "behavioral"[All Fields] OR "behavioural"[All Fields] OR "behavior s"[All Fields] OR "behaviorally"[All Fields] OR "behaviour"[All Fields] OR "behaviourally"[All Fields] OR "behaviours"[All Fields] OR "behaviors"[All Fields] OR "pattern"[All Fields] OR "pattern s"[All Fields] OR "patternability"[All Fields] OR "patternable"[All Fields] OR "patterned"[All Fields] OR "patterning"[All Fields] OR "patternings"[All Fields] OR "patterns"[All Fields]) AND ("change"[All Fields] OR "changed"[All Fields] OR "changes"[All Fields] OR "changing"[All Fields] OR "changings"[All Fields]) AND ("theorie"[All Fields] OR "theories"[All Fields] OR "theory"[All Fields] OR "theory s"[All Fields]))) AND 2019/01/01:2023/12/31[Date - Publication])) AND (2019:2023[pdat])	
13	health action process approach or hapa	from 2019 - 2023	((("healthaction"[Journal] OR ("health"[All Fields] AND "action"[All Fields]) OR "health action"[All Fields]) AND ("process"[All Fields] OR "processe"[All Fields] OR "processed"[All Fields] OR "processes"[All Fields] OR "processing"[All Fields] OR "processings"[All Fields]) AND ("approach"[All Fields] OR "approach s"[All Fields] OR "approachability"[All Fields] OR "approachable"[All Fields] OR "approache"[All Fields] OR "approached"[All Fields] OR "approaches"[All Fields] OR "approaching"[All Fields] OR "approachs"[All Fields])) OR "hapa"[All Fields]) AND (2019:2023[pdat])	2,518
12	theoretical domains framework or TDF	from 2019 - 2023	((("theoretic"[All Fields] OR "theoretical"[All Fields] OR "theoretically"[All Fields]) AND ("domain s"[All Fields] OR "domains"[All Fields] OR "protein domains"[MeSH Terms] OR ("protein"[All Fields] AND "domains"[All Fields]) OR "protein domains"[All Fields] OR "domain"[All Fields]) AND ("framework"[All Fields] OR "framework s"[All Fields] OR "frameworks"[All Fields])) OR "TDF"[All Fields]) AND (2019:2023[pdat])	3,151
11	behaviour change wheel or behavior change wheel BCW	from 2019 - 2023	((("behav change"[Journal] OR ("behaviour"[All Fields] AND "change"[All Fields]) OR "behaviour change"[All Fields]) AND ("wheel"[All Fields] OR "wheeled"[All Fields] OR "wheeling"[All Fields] OR "wheels"[All Fields])) OR (("behavior"[MeSH Terms] OR "behavior"[All Fields] OR "behavioral"[All Fields] OR "behavioural"[All Fields] OR "behavior s"[All Fields] OR "behaviorally"[All Fields] OR "behaviour"[All Fields] OR "behaviourally"[All Fields] OR "behaviours"[All Fields] OR "behaviors"[All Fields] OR "pattern"[All Fields] OR "pattern s"[All Fields] OR "patternability"[All Fields] OR "patternable"[All Fields] OR "patterned"[All Fields] OR "patterning"[All Fields] OR "patternings"[All Fields] OR "patterns"[All Fields]) AND ("change"[All Fields] OR "changed"[All Fields] OR "changes"[All Fields] OR "changing"[All Fields] OR "changings"[All Fields]) AND ("wheel"[All Fields] OR "wheeled"[All Fields] OR "wheeling"[All Fields] OR "wheels"[All Fields]) AND "BCW"[All Fields])) AND (2019:2023[pdat])	379
10	com-b model or com-b or com b or com-b	from	(("com-b"[All Fields] AND ("model"[All Fields] OR "model s"[All Fields] OR "modeled"[All Fields] OR "modeler"[All Fields]	52,684

	framework	2019 - 2023	OR "modeler s"[All Fields] OR "modelers"[All Fields] OR "modeling"[All Fields] OR "modelings"[All Fields] OR "modelization"[All Fields] OR "modelizations"[All Fields] OR "modelize"[All Fields] OR "modelized"[All Fields] OR "modelled"[All Fields] OR "modeller"[All Fields] OR "modellers"[All Fields] OR "modelling"[All Fields] OR "modellings"[All Fields] OR "models"[All Fields])) OR "com-b"[All Fields] OR ("com"[All Fields] AND "b"[All Fields]) OR ("com-b"[All Fields] AND ("framework"[All Fields] OR "framework s"[All Fields] OR "frameworks"[All Fields]))) AND (2019:2023[pdat])	
9	protection motivation theory or pmt	from 2019 - 2023	((("protect"[All Fields] OR "protected"[All Fields] OR "protecting"[All Fields] OR "protection"[All Fields] OR "protections"[All Fields] OR "protective agents"[Pharmacological Action] OR "protective agents"[MeSH Terms] OR ("protective"[All Fields] AND "agents"[All Fields]) OR "protective agents"[All Fields] OR "protectant"[All Fields] OR "protectants"[All Fields] OR "protective"[All Fields] OR "protectively"[All Fields] OR "protectiveness"[All Fields] OR "protectives"[All Fields] OR "protects"[All Fields]) AND ("motivate"[All Fields] OR "motivated"[All Fields] OR "motivates"[All Fields] OR "motivating"[All Fields] OR "motivation"[MeSH Terms] OR "motivation"[All Fields] OR "motivations"[All Fields] OR "motive"[All Fields] OR "motivational"[All Fields] OR "motivator"[All Fields] OR "motivators"[All Fields] OR "motives"[All Fields]) AND ("theorie"[All Fields] OR "theories"[All Fields] OR "theory"[All Fields] OR "theory s"[All Fields])) OR "pmt"[All Fields]) AND (2019:2023[pdat])	1,405
8	social cognitive theory or sct or social-cognitive theory or social cognitive learning theory	from 2019 - 2023	("psychological theory"[MeSH Terms] OR ("psychological"[All Fields] AND "theory"[All Fields]) OR "psychological theory"[All Fields] OR ("social"[All Fields] AND "cognitive"[All Fields] AND "theory"[All Fields]) OR "social cognitive theory"[All Fields] OR "sct"[All Fields] OR ("psychological theory"[MeSH Terms] OR ("psychological"[All Fields] AND "theory"[All Fields]) OR "psychological theory"[All Fields] OR ("social"[All Fields] AND "cognitive"[All Fields] AND "theory"[All Fields]) OR "social cognitive theory"[All Fields]) OR (("social behavior"[MeSH Terms] OR ("social"[All Fields] AND "behavior"[All Fields]) OR "social behavior"[All Fields] OR "sociality"[All Fields] OR "social"[All Fields] OR "socialisation"[All Fields] OR "socialization"[MeSH Terms] OR "socialization"[All Fields] OR "socialise"[All Fields] OR "socialised"[All Fields] OR "socialising"[All Fields] OR "socialities"[All Fields] OR "socializations"[All Fields] OR "socialize"[All Fields] OR "socialized"[All Fields] OR "socializers"[All Fields] OR "socializes"[All Fields] OR "socializing"[All Fields] OR "socially"[All Fields] OR "socials"[All Fields]) AND ("cognition"[MeSH Terms] OR "cognition"[All Fields] OR "cognitions"[All Fields] OR "cognitive"[All Fields] OR "cognitively"[All Fields] OR "cognitives"[All Fields]) AND ("learning"[MeSH Terms] OR "learning"[All Fields] OR "learn"[All Fields] OR "learned"[All Fields] OR "learning s"[All Fields] OR "learnings"[All Fields] OR "learns"[All Fields]) AND ("theorie"[All Fields] OR "theories"[All Fields] OR "theory"[All Fields] OR "theory s"[All Fields]))) AND (2019:2023[pdat])	24,967
7	transtheoretical model or trans-theoretical model or stages of change	from 2019 - 2023	("transtheoretical model"[MeSH Terms] OR ("transtheoretical"[All Fields] AND "model"[All Fields]) OR "transtheoretical model"[All Fields] OR ("trans-theoretical"[All Fields] AND ("model"[All Fields] OR "model s"[All Fields] OR "modeled"[All Fields] OR "modeler"[All Fields] OR "modeler s"[All Fields] OR "modelers"[All Fields] OR "modeling"[All Fields] OR "modelings"[All Fields] OR "modelization"[All Fields] OR "modelizations"[All Fields] OR "modelize"[All Fields] OR "modelized"[All Fields] OR "modelled"[All Fields] OR "modeller"[All Fields] OR "modellers"[All Fields] OR "modelling"[All Fields] OR "modellings"[All Fields] OR "models"[All Fields])) OR ("transtheoretical model"[MeSH Terms] OR ("transtheoretical"[All Fields] AND "model"[All Fields]) OR "transtheoretical model"[All Fields] OR ("stages"[All Fields] AND "change"[All Fields]) OR "stages of change"[All Fields])) AND (2019:2023[pdat])	8,125
6	theory of reasoned action or TRA	from 2019 - 2023	("theory of planned behavior"[MeSH Terms] OR ("theory"[All Fields] AND "planned"[All Fields] AND "behavior"[All Fields]) OR "theory of planned behavior"[All Fields] OR ("theory"[All Fields] AND "reasoned"[All Fields] AND "action"[All Fields]) OR "theory of reasoned action"[All Fields] OR "TRA"[All Fields]) AND (2019:2023[pdat])	3,326
5	theory of planned behavior or theory of planned behaviour or tpb	from 2019 - 2023	("theory of planned behavior"[MeSH Terms] OR ("theory"[All Fields] AND "planned"[All Fields] AND "behavior"[All Fields]) OR "theory of planned behavior"[All Fields] OR ("theory of planned behavior"[MeSH Terms] OR ("theory"[All Fields] AND "planned"[All Fields] AND "behavior"[All Fields]) OR "theory of planned behavior"[All Fields] OR ("theory"[All Fields] AND "planned"[All Fields] AND "behaviour"[All Fields]) OR "theory of planned behaviour"[All Fields]) OR "tpb"[All Fields]) AND (2019:2023[pdat])	2,359

4	health belief model or health belief theory or hbm	from 2019 - 2023	("health belief model"[MeSH Terms] OR ("health"[All Fields] AND "belief"[All Fields] AND "model"[All Fields]) OR "health belief model"[All Fields] OR (("health"[MeSH Terms] OR "health"[All Fields] OR "health s"[All Fields] OR "healthful"[All Fields] OR "healthfulness"[All Fields] OR "healths"[All Fields]) AND ("belief s"[All Fields] OR "culture"[MeSH Terms] OR "culture"[All Fields] OR "belief"[All Fields] OR "beliefs"[All Fields]) AND ("theorie"[All Fields] OR "theories"[All Fields] OR "theory"[All Fields] OR "theory s"[All Fields])) OR "hbm"[All Fields]) AND (2019:2023[pdat])	6,590
3	behaviour theory or behavioural theory or behavioural change theory	from 2019 - 2023	((("behavior"[MeSH Terms] OR "behavior"[All Fields] OR "behavioral"[All Fields] OR "behavioural"[All Fields] OR "behavior s"[All Fields] OR "behaviorally"[All Fields] OR "behaviour"[All Fields] OR "behaviourally"[All Fields] OR "behaviours"[All Fields] OR "behaviors"[All Fields] OR "pattern"[All Fields] OR "pattern s"[All Fields] OR "patternability"[All Fields] OR "patternable"[All Fields] OR "patterned"[All Fields] OR "patterning"[All Fields] OR "patternings"[All Fields] OR "patterns"[All Fields]) AND ("theorie"[All Fields] OR "theories"[All Fields] OR "theory"[All Fields] OR "theory s"[All Fields])) OR (("behavior"[MeSH Terms] OR "behavior"[All Fields] OR "behavioral"[All Fields] OR "behavioural"[All Fields] OR "behavior s"[All Fields] OR "behaviorally"[All Fields] OR "behaviour"[All Fields] OR "behaviourally"[All Fields] OR "behaviours"[All Fields] OR "behaviors"[All Fields] OR "pattern"[All Fields] OR "pattern s"[All Fields] OR "patternability"[All Fields] OR "patternable"[All Fields] OR "patterned"[All Fields] OR "patterning"[All Fields] OR "patternings"[All Fields] OR "patterns"[All Fields]) AND ("theorie"[All Fields] OR "theories"[All Fields] OR "theory"[All Fields] OR "theory s"[All Fields])) OR (("behavior"[MeSH Terms] OR "behavior"[All Fields] OR "behavioral"[All Fields] OR "behavioural"[All Fields] OR "behavior s"[All Fields] OR "behaviorally"[All Fields] OR "behaviour"[All Fields] OR "behaviourally"[All Fields] OR "behaviours"[All Fields] OR "behaviors"[All Fields] OR "pattern"[All Fields] OR "pattern s"[All Fields] OR "patternability"[All Fields] OR "patternable"[All Fields] OR "patterned"[All Fields] OR "patterning"[All Fields] OR "patternings"[All Fields] OR "patterns"[All Fields]) AND ("change"[All Fields] OR "changed"[All Fields] OR "changes"[All Fields] OR "changing"[All Fields] OR "changings"[All Fields]) AND ("theorie"[All Fields] OR "theories"[All Fields] OR "theory"[All Fields] OR "theory s"[All Fields]))) AND (2019:2023[pdat])	44,041
2	covid-19 or coronavirus or 2019-ncov or sars-cov-2 or cov-19	from 2019 - 2023	("covid 19"[All Fields] OR "covid 19"[MeSH Terms] OR "covid 19 vaccines"[All Fields] OR "covid 19 vaccines"[MeSH Terms] OR "covid 19 serotherapy"[All Fields] OR "covid 19 nucleic acid testing"[All Fields] OR "covid 19 nucleic acid testing"[MeSH Terms] OR "covid 19 serological testing"[All Fields] OR "covid 19 serological testing"[MeSH Terms] OR "covid 19 testing"[All Fields] OR "covid 19 testing"[MeSH Terms] OR "sars cov 2"[All Fields] OR "sars cov 2"[MeSH Terms] OR "severe acute respiratory syndrome coronavirus 2"[All Fields] OR "ncov"[All Fields] OR "2019 ncov"[All Fields] OR (("coronavirus"[MeSH Terms] OR "coronavirus"[All Fields] OR "cov"[All Fields]) AND 2019/11/01:3000/12/31[Date - Publication]) OR ("coronavirus"[MeSH Terms] OR "coronavirus"[All Fields] OR "coronaviruses"[All Fields]) OR ("sars cov 2"[MeSH Terms] OR "sars cov 2"[All Fields] OR "2019 ncov"[All Fields]) OR ("sars cov 2"[MeSH Terms] OR "sars cov 2"[All Fields] OR "sars cov 2"[All Fields]) OR "cov-19"[All Fields]) AND (2019:2023[pdat])	348,758
1	healthcare workers or healthcare professional or healthcare provider or healthcare personnel or doctor or nurse	from 2019 - 2023	("health personnel"[MeSH Terms] OR ("health"[All Fields] AND "personnel"[All Fields]) OR "health personnel"[All Fields] OR ("healthcare"[All Fields] AND "workers"[All Fields]) OR "healthcare workers"[All Fields] OR ("health personnel"[MeSH Terms] OR ("health"[All Fields] AND "personnel"[All Fields]) OR "health personnel"[All Fields] OR ("healthcare"[All Fields] AND "professional"[All Fields]) OR "healthcare professional"[All Fields]) OR ("health personnel"[MeSH Terms] OR ("health"[All Fields] AND "personnel"[All Fields]) OR "health personnel"[All Fields] OR ("healthcare"[All Fields] AND "provider"[All Fields]) OR "healthcare provider"[All Fields]) OR (("delivery of health care"[MeSH Terms] OR ("delivery"[All Fields] AND "health"[All Fields] AND "care"[All Fields]) OR "delivery of health care"[All Fields] OR "healthcare"[All Fields] OR "healthcare s"[All Fields] OR "healthcares"[All Fields]) AND ("occupational groups"[MeSH Terms] OR ("occupational"[All Fields] AND "groups"[All Fields]) OR "occupational groups"[All Fields] OR "personnel"[All Fields] OR "personnel s"[All Fields] OR "personnels"[All Fields])) OR ("doctor s"[All Fields] OR "doctoral"[All Fields] OR "doctorally"[All Fields] OR "doctorate"[All Fields] OR "doctorates"[All Fields] OR "doctoring"[All Fields] OR "physicians"[MeSH Terms] OR "physicians"[All Fields] OR "doctor"[All Fields] OR "doctors"[All Fields]) OR ("nurse s"[All Fields] OR "nurses"[MeSH Terms] OR "nurses"[All Fields] OR "nurse"[All Fields] OR "nurses s"[All Fields])) AND (2019:2023[pdat])	319,530

ProQuest search strategy			
Set#	Searched for	Databases	Results
S1	abstract(healthcare workers OR healthcare professional OR healthcare provider OR healthcare personnel OR doctor OR nurse) AND pd(>20190131)	APA PsycArticles®, APA PsycInfo®, Art, Design & Architecture Collection, British Periodicals, Coronavirus Research Database, Early Modern Books, Ebook Central, Entertainment Industry Magazine Archive, Humanities Index, Periodicals Archive Online, ProQuest Historical Newspapers: The Guardian and The Observer, ProQuest One Academic, PTSDpubs, SciTech Premium Collection, Social Science Premium Collection, Sports Medicine & Education Index, The Vogue Archive, The Women's Wear Daily Archive	282500
S2	abstract(covid-19 OR coronavirus OR 2019-ncov OR sars-cov-2 OR cov-19) AND pd(>20190131)	APA PsycArticles®, APA PsycInfo®, Art, Design & Architecture Collection, British Periodicals, Coronavirus Research Database, Early Modern Books, Ebook Central, Entertainment Industry Magazine Archive, Humanities Index, Periodicals Archive Online, ProQuest Historical Newspapers: The Guardian and The Observer, ProQuest One Academic, PTSDpubs, SciTech Premium Collection, Social Science Premium Collection, Sports Medicine & Education Index, The Vogue Archive, The Women's Wear Daily Archive	638169
S3	abstract(behaviour theory OR behavioural theory OR theory of behaviour OR behavioural change theory OR theory of behaviour change) AND pd(>20190131)	APA PsycArticles®, APA PsycInfo®, Art, Design & Architecture Collection, British Periodicals, Coronavirus Research Database, Early Modern Books, Ebook Central, Entertainment Industry Magazine Archive, Humanities Index, Periodicals Archive Online, ProQuest Historical Newspapers: The Guardian and The Observer, ProQuest One Academic, PTSDpubs, SciTech Premium Collection, Social Science Premium Collection, Sports Medicine & Education Index, The Vogue Archive, The Women's Wear Daily Archive	108468
S4	abstract(health belief model OR health belief theory OR hbm) AND pd(>20190131)	APA PsycArticles®, APA PsycInfo®, Art, Design & Architecture Collection, British Periodicals, Coronavirus Research Database, Early Modern Books, Ebook Central, Entertainment Industry Magazine Archive, Humanities Index, Periodicals Archive Online, ProQuest Historical Newspapers: The Guardian and The Observer, ProQuest One Academic, PTSDpubs, SciTech Premium Collection, Social Science Premium Collection, Sports Medicine & Education Index, The Vogue Archive, The Women's Wear Daily Archive	12033
S5	abstract(theory of planned behavior OR theory of planned behaviour OR tpb) AND pd(>20190131)	APA PsycArticles®, APA PsycInfo®, Art, Design & Architecture Collection, British Periodicals, Coronavirus Research Database, Early Modern Books, Ebook Central, Entertainment Industry Magazine Archive, Humanities Index, Periodicals Archive Online, ProQuest Historical Newspapers: The Guardian and The Observer, ProQuest One Academic, PTSDpubs, SciTech Premium Collection, Social Science Premium Collection, Sports Medicine & Education Index, The Vogue Archive, The Women's Wear Daily Archive	8984
S6	abstract(theory of reasoned action OR TRA) AND pd(>20190131)	APA PsycArticles®, APA PsycInfo®, Art, Design & Architecture Collection, British Periodicals, Coronavirus Research Database, Early Modern Books, Ebook Central, Entertainment Industry Magazine Archive, Humanities Index, Periodicals Archive Online, ProQuest Historical Newspapers: The Guardian and The Observer, ProQuest One Academic, PTSDpubs, SciTech Premium Collection, Social Science Premium Collection, Sports Medicine & Education Index, The Vogue Archive, The Women's Wear Daily Archive	4829
S7	abstract(transtheoretical model OR trans-theoretical model OR stages of change) AND pd(>20190131)	APA PsycArticles®, APA PsycInfo®, Art, Design & Architecture Collection, British Periodicals, Coronavirus Research Database, Early Modern Books, Ebook Central, Entertainment Industry Magazine Archive, Humanities Index, Periodicals Archive Online, ProQuest Historical Newspapers: The Guardian and The Observer, ProQuest One Academic, PTSDpubs, SciTech Premium Collection, Social Science Premium Collection, Sports Medicine & Education Index, The Vogue Archive, The Women's Wear Daily Archive	116012

S8	abstract(social cognitive theory OR sct OR social-cognitive theory OR social cognitive learning theory) AND pd(>20190131)	APA PsycArticles®, APA PsycInfo®, Art, Design & Architecture Collection, British Periodicals, Coronavirus Research Database, Early Modern Books, Ebook Central, Entertainment Industry Magazine Archive, Humanities Index, Periodicals Archive Online, ProQuest Historical Newspapers: The Guardian and The Observer, ProQuest One Academic, PTSDpubs, SciTech Premium Collection, Social Science Premium Collection, Sports Medicine & Education Index, The Vogue Archive, The Women's Wear Daily Archive	15982
S9	abstract(protection motivation theory OR pmt) AND pd(>20190131)	APA PsycArticles®, APA PsycInfo®, Art, Design & Architecture Collection, British Periodicals, Coronavirus Research Database, Early Modern Books, Ebook Central, Entertainment Industry Magazine Archive, Humanities Index, Periodicals Archive Online, ProQuest Historical Newspapers: The Guardian and The Observer, ProQuest One Academic, PTSDpubs, SciTech Premium Collection, Social Science Premium Collection, Sports Medicine & Education Index, The Vogue Archive, The Women's Wear Daily Archive	2728
S10	abstract(com-b model OR com-b OR com b OR com-b framework) AND pd(>20190131)	APA PsycArticles®, APA PsycInfo®, Art, Design & Architecture Collection, British Periodicals, Coronavirus Research Database, Early Modern Books, Ebook Central, Entertainment Industry Magazine Archive, Humanities Index, Periodicals Archive Online, ProQuest Historical Newspapers: The Guardian and The Observer, ProQuest One Academic, PTSDpubs, SciTech Premium Collection, Social Science Premium Collection, Sports Medicine & Education Index, The Vogue Archive, The Women's Wear Daily Archive	5229
S11	abstract(behaviour change wheel OR behavior change wheel BCW) AND pd(>20190131)	APA PsycArticles®, APA PsycInfo®, Art, Design & Architecture Collection, British Periodicals, Coronavirus Research Database, Early Modern Books, Ebook Central, Entertainment Industry Magazine Archive, Humanities Index, Periodicals Archive Online, ProQuest Historical Newspapers: The Guardian and The Observer, ProQuest One Academic, PTSDpubs, SciTech Premium Collection, Social Science Premium Collection, Sports Medicine & Education Index, The Vogue Archive, The Women's Wear Daily Archive	1287
S12	abstract(theoretical domains framework OR TDF) AND pd(>20190131)	APA PsycArticles®, APA PsycInfo®, Art, Design & Architecture Collection, British Periodicals, Coronavirus Research Database, Early Modern Books, Ebook Central, Entertainment Industry Magazine Archive, Humanities Index, Periodicals Archive Online, ProQuest Historical Newspapers: The Guardian and The Observer, ProQuest One Academic, PTSDpubs, SciTech Premium Collection, Social Science Premium Collection, Sports Medicine & Education Index, The Vogue Archive, The Women's Wear Daily Archive	7880
S13	abstract(health action process approach OR hapa) AND pd(>20190131)	APA PsycArticles®, APA PsycInfo®, Art, Design & Architecture Collection, British Periodicals, Coronavirus Research Database, Early Modern Books, Ebook Central, Entertainment Industry Magazine Archive, Humanities Index, Periodicals Archive Online, ProQuest Historical Newspapers: The Guardian and The Observer, ProQuest One Academic, PTSDpubs, SciTech Premium Collection, Social Science Premium Collection, Sports Medicine & Education Index, The Vogue Archive, The Women's Wear Daily Archive	4211
S14	(abstract(healthcare workers OR healthcare professional OR healthcare provider OR healthcare personnel OR doctor OR nurse) AND pd(>20190131)) AND (abstract(covid-19 OR coronavirus OR 2019-ncov OR sars-cov-2 OR cov-19) AND pd(>20190131)) AND (abstract(behaviour theory OR behavioural theory OR theory of behaviour OR behavioural change theory OR theory of behaviour change) AND pd(>20190131))	APA PsycArticles®, APA PsycInfo®, Art, Design & Architecture Collection, British Periodicals, Coronavirus Research Database, Early Modern Books, Ebook Central, Entertainment Industry Magazine Archive, Humanities Index, Periodicals Archive Online, ProQuest Historical Newspapers: The Guardian and The Observer, ProQuest One Academic, PTSDpubs, SciTech Premium Collection, Social Science Premium Collection, Sports Medicine & Education Index, The Vogue Archive, The Women's Wear Daily Archive<br>These databases are searched for part of your query.	203

S15	(abstract(healthcare workers OR healthcare professional OR healthcare provider OR healthcare personnel OR doctor OR nurse) AND pd(>20190131)) AND (abstract(covid-19 OR coronavirus OR 2019-ncov OR sars-cov-2 OR cov-19) AND pd(>20190131)) AND (abstract(health belief model OR health belief theory OR hbm) AND pd(>20190131))	APA PsycArticles®, APA PsycInfo®, Art, Design & Architecture Collection, British Periodicals, Coronavirus Research Database, Early Modern Books, Ebook Central, Entertainment Industry Magazine Archive, Humanities Index, Periodicals Archive Online, ProQuest Historical Newspapers: The Guardian and The Observer, ProQuest One Academic, PTSDpubs, SciTech Premium Collection, Social Science Premium Collection, Sports Medicine & Education Index, The Vogue Archive, The Women's Wear Daily Archive<br>These databases are searched for part of your query.	205
S16	(abstract(healthcare workers OR healthcare professional OR healthcare provider OR healthcare personnel OR doctor OR nurse) AND pd(>20190131)) AND (abstract(covid-19 OR coronavirus OR 2019-ncov OR sars-cov-2 OR cov-19) AND pd(>20190131)) AND (abstract(theory of planned behavior OR theory of planned behaviour OR tpb) AND pd(>20190131))	APA PsycArticles®, APA PsycInfo®, Art, Design & Architecture Collection, British Periodicals, Coronavirus Research Database, Early Modern Books, Ebook Central, Entertainment Industry Magazine Archive, Humanities Index, Periodicals Archive Online, ProQuest Historical Newspapers: The Guardian and The Observer, ProQuest One Academic, PTSDpubs, SciTech Premium Collection, Social Science Premium Collection, Sports Medicine & Education Index, The Vogue Archive, The Women's Wear Daily Archive<br>These databases are searched for part of your query.	39
S17	(abstract(healthcare workers OR healthcare professional OR healthcare provider OR healthcare personnel OR doctor OR nurse) AND pd(>20190131)) AND (abstract(covid-19 OR coronavirus OR 2019-ncov OR sars-cov-2 OR cov-19) AND pd(>20190131)) AND (abstract(theory of reasoned action OR TRA) AND pd(>20190131))	APA PsycArticles®, APA PsycInfo®, Art, Design & Architecture Collection, British Periodicals, Coronavirus Research Database, Early Modern Books, Ebook Central, Entertainment Industry Magazine Archive, Humanities Index, Periodicals Archive Online, ProQuest Historical Newspapers: The Guardian and The Observer, ProQuest One Academic, PTSDpubs, SciTech Premium Collection, Social Science Premium Collection, Sports Medicine & Education Index, The Vogue Archive, The Women's Wear Daily Archive<br>These databases are searched for part of your query.	8
S18	(abstract(healthcare workers OR healthcare professional OR healthcare provider OR healthcare personnel OR doctor OR nurse) AND pd(>20190131)) AND (abstract(covid-19 OR coronavirus OR 2019-ncov OR sars-cov-2 OR cov-19) AND pd(>20190131)) AND (abstract(transtheoretical model OR trans-theoretical model OR stages of change) AND pd(>20190131))	APA PsycArticles®, APA PsycInfo®, Art, Design & Architecture Collection, British Periodicals, Coronavirus Research Database, Early Modern Books, Ebook Central, Entertainment Industry Magazine Archive, Humanities Index, Periodicals Archive Online, ProQuest Historical Newspapers: The Guardian and The Observer, ProQuest One Academic, PTSDpubs, SciTech Premium Collection, Social Science Premium Collection, Sports Medicine & Education Index, The Vogue Archive, The Women's Wear Daily Archive<br>These databases are searched for part of your query.	237
S19	(abstract(healthcare workers OR healthcare professional OR healthcare provider OR healthcare personnel OR doctor OR nurse) AND pd(>20190131)) AND (abstract(covid-19 OR coronavirus OR 2019-ncov OR sars-cov-2 OR cov-19) AND pd(>20190131)) AND (abstract(social cognitive theory OR sct OR social-cognitive theory OR social cognitive	APA PsycArticles®, APA PsycInfo®, Art, Design & Architecture Collection, British Periodicals, Coronavirus Research Database, Early Modern Books, Ebook Central, Entertainment Industry Magazine Archive, Humanities Index, Periodicals Archive Online, ProQuest Historical Newspapers: The Guardian and The Observer, ProQuest One Academic, PTSDpubs, SciTech Premium Collection, Social Science Premium Collection, Sports Medicine & Education Index, The Vogue Archive, The Women's Wear Daily Archive<br>These databases are searched for part of your query.	24

	learning theory) AND pd(>20190131))		
S20	(abstract(healthcare workers OR healthcare professional OR healthcare provider OR healthcare personnel OR doctor OR nurse) AND pd(>20190131)) AND (abstract(covid-19 OR coronavirus OR 2019-ncov OR sars-cov-2 OR cov-19) AND pd(>20190131)) AND (abstract(protection motivation theory OR pmt) AND pd(>20190131))	APA PsycArticles®, APA PsycInfo®, Art, Design & Architecture Collection, British Periodicals, Coronavirus Research Database, Early Modern Books, Ebook Central, Entertainment Industry Magazine Archive, Humanities Index, Periodicals Archive Online, ProQuest Historical Newspapers: The Guardian and The Observer, ProQuest One Academic, PTSDpubs, SciTech Premium Collection, Social Science Premium Collection, Sports Medicine & Education Index, The Vogue Archive, The Women's Wear Daily Archive<br>These databases are searched for part of your query.	14
S21	(abstract(healthcare workers OR healthcare professional OR healthcare provider OR healthcare personnel OR doctor OR nurse) AND pd(>20190131)) AND (abstract(covid-19 OR coronavirus OR 2019-ncov OR sars-cov-2 OR cov-19) AND pd(>20190131)) AND (abstract(com-b model OR com-b OR com b OR com-b framework) AND pd(>20190131))	APA PsycArticles®, APA PsycInfo®, Art, Design & Architecture Collection, British Periodicals, Coronavirus Research Database, Early Modern Books, Ebook Central, Entertainment Industry Magazine Archive, Humanities Index, Periodicals Archive Online, ProQuest Historical Newspapers: The Guardian and The Observer, ProQuest One Academic, PTSDpubs, SciTech Premium Collection, Social Science Premium Collection, Sports Medicine & Education Index, The Vogue Archive, The Women's Wear Daily Archive<br>These databases are searched for part of your query.	33
S22	(abstract(healthcare workers OR healthcare professional OR healthcare provider OR healthcare personnel OR doctor OR nurse) AND pd(>20190131)) AND (abstract(covid-19 OR coronavirus OR 2019-ncov OR sars-cov-2 OR cov-19) AND pd(>20190131)) AND (abstract(behaviour change wheel OR behavior change wheel BCW) AND pd(>20190131))	APA PsycArticles®, APA PsycInfo®, Art, Design & Architecture Collection, British Periodicals, Coronavirus Research Database, Early Modern Books, Ebook Central, Entertainment Industry Magazine Archive, Humanities Index, Periodicals Archive Online, ProQuest Historical Newspapers: The Guardian and The Observer, ProQuest One Academic, PTSDpubs, SciTech Premium Collection, Social Science Premium Collection, Sports Medicine & Education Index, The Vogue Archive, The Women's Wear Daily Archive<br>These databases are searched for part of your query.	10
S23	(abstract(healthcare workers OR healthcare professional OR healthcare provider OR healthcare personnel OR doctor OR nurse) AND pd(>20190131)) AND (abstract(covid-19 OR coronavirus OR 2019-ncov OR sars-cov-2 OR cov-19) AND pd(>20190131)) AND (abstract(theoretical domains framework OR TDF) AND pd(>20190131))	APA PsycArticles®, APA PsycInfo®, Art, Design & Architecture Collection, British Periodicals, Coronavirus Research Database, Early Modern Books, Ebook Central, Entertainment Industry Magazine Archive, Humanities Index, Periodicals Archive Online, ProQuest Historical Newspapers: The Guardian and The Observer, ProQuest One Academic, PTSDpubs, SciTech Premium Collection, Social Science Premium Collection, Sports Medicine & Education Index, The Vogue Archive, The Women's Wear Daily Archive<br>These databases are searched for part of your query.	25
S24	(abstract(healthcare workers OR healthcare professional OR healthcare provider OR healthcare personnel OR doctor OR nurse) AND pd(>20190131)) AND (abstract(covid-19 OR coronavirus OR 2019-ncov OR sars-cov-2 OR cov-19) AND pd(>20190131)) AND (abstract(health action process approach OR hapa) AND pd(>20190131))	APA PsycArticles®, APA PsycInfo®, Art, Design & Architecture Collection, British Periodicals, Coronavirus Research Database, Early Modern Books, Ebook Central, Entertainment Industry Magazine Archive, Humanities Index, Periodicals Archive Online, ProQuest Historical Newspapers: The Guardian and The Observer, ProQuest One Academic, PTSDpubs, SciTech Premium Collection, Social Science Premium Collection, Sports Medicine & Education Index, The Vogue Archive, The Women's Wear Daily Archive<br>These databases are searched for part of your query.	49

Supplementary File 3: Characteristics of excluded studies (Ordered alphabetically)

**Study**	**Reasons for exclusion**
Ahmad et al. (2020)	Mixed sample with no separate outcomes for HCWs.
Arslanca et al. (2021)	No use of behaviour change theory, model or framework
Cui et al. (2022)	Limited focus on IPC behaviours
Fauk et al. (2022)	Unrelated outcome (preventive behaviours in relation to social life)
Gammon and Hunt (2020)	No use of behaviour change theory, model or framework
Girma et al. (2020)	No use of behaviour change theory, model or framework
Hwang and Kim (2022)	Limited focus on IPC behaviours
Khalid et al. (2021)	Mixed sample with no separate outcomes for HCWs
Kierkegaard et al. (2021)	Not a primary study
Kim et al. (2020)	Limited focus on IPC behaviours
Lyu et al. (2021)	Unrelated outcome (development and testing of model)
Matthews et al. (2021)	Not focused on HCWs
Maykrantz et al. (2021)	Unrelated outcome (development and testing of model)

Minuye et al. (2021)	Limited focus on IPC
Moss et al. (2023)	Mixed sample with no separate outcomes for HCWs.
Nahidi et al. (2022)	Limited focus on IPC behaviours
Pesiridis et al. (2021)	Limited focus on IPC behaviours
Rahmatpour et al. (2020)	Limited focus on IPC behaviours
Sharma et al. (2020)	Limited focus on IPC behaviours
Storr-Mathis (2021)	Mixed sample with no separate outcomes for HCWs
Tong et al. (2022)	Limited focus on IPC behaviours
Zewude et al. (2021)	No use of behaviour change theory, model or framework
Zhu et al. (2021)	Limited focus on IPC behaviours

HCWs: healthcare workers; IPC: infection prevention and control

Reference (Excluded studies)

Ahmad M, Iram K and Jabeen G (2020) Perception-based influence factors of intention to adopt COVID-19 epidemic prevention in China. *Environmental Research* 190: 109995. DOI: https://doi.org/10.1016/j.envres.2020.109995.

Arslanca T, Fidan C, Daggez M, et al. (2021) Knowledge, preventive behaviors and risk perception of the COVID-19 pandemic: a cross-sectional study in Turkish health care workers. *PLOS ONE* 16(4): e0250017. DOI: https://doi.org/10.1371/journal.pone.0250017.

Cui F, Jin Y, Wu H, et al. (2022) Behavioral intentions and factors influencing nurses' care of COVID-19 patients: a cross-sectional study. *Frontiers in Public Health* 10: 914599. DOI: https://doi.org/10.3389/fpubh.2022.914599.

Fauk NK, Seran AL, Raymond C, et al. (2022) Why do we not follow lifesaving rules? factors affecting nonadherence to COVID-19 prevention guidelines in Indonesia: healthcare professionals' perspectives. *International Journal of Environmental Research and Public Health* 19(14): 8502. DOI: https://doi.org/10.3390/ijerph19148502.

Gammon J and Hunt J (2020) COVID-19 and hand hygiene: the vital importance of hand drying. British Journal of Nursing 29(17): 1003–1006. DOI: https://doi.org/10.12968/bjon.2020.29.17.1003.

Girma S, Agenagnew L, Beressa G, et al. (2020) Risk perception and precautionary health behavior toward COVID-19 among health professionals working in selected public university hospitals in Ethiopia. *PLOS ONE* 15(10): e0241101. DOI: https://doi.org/10.1371/journal.pone.0241101.

Hwang A and Kim D (2022) Factors influencing nurses' intent to provide care involved in coronavirus disease 2019: theory of planned behaviour perspectives. *Journal of Clinical Nursing*. DOI: https://doi.org/10.1111/jocn.16468.

Khalid AM, Noor I, Naiyar I, et al. (2021) Behavioral trends of medical institution employees in adhering to covid-19 preventive strategies. *Rawal Medical Journal* 46(3): 698–701. Available at: https://pesquisa.bvsalud.org/global-literature-on-novel-coronavirus-2019-ncov/resource/en/covidwho-1357887 (accessed 15 May 2023).

Kierkegaard P, Hicks T, Allen AM, et al. (2021) Strategies to implement SARS-CoV-2 point-of-care testing into primary care settings: a qualitative secondary analysis guided by the Behaviour Change Wheel. *Implement Sci Commun* 2(1): 139. DOI: https://doi.org/10.1186/s43058-021-00242-6.

Kim Y-J, Lee S-Y and Cho J-H (2020) A study on the job retention intention of nurses based on social support in the COVID-19 situation. *Sustainability* 12(18): 7276. DOI: https://doi.org/10.3390/su12187276.

Lyu X, Hu J, Xu X, et al. (2021) Factors influencing risk perception and nosocomial infection prevention practices of frontline nurses during the COVID-19 pandemic. *BMC Nursing* 20(1): 1–17. DOI: https://doi.org/10.1186/s12912-021-00591-6.

Matthews VS, Stough-Hunter A and Marazita JM (2021) Attitudes towards social distancing in response to COVID-19. *Public Health Nursing* 38(6): 1019–1029. DOI: https://doi.org/10.1111/phn.12954.

Maykrantz SA, Langlinais LA, Houghton JD, et al. (2021) Self-leadership and psychological capital as key cognitive resources for shaping health-protective behaviors during the COVID-19 pandemic. *Administrative Sciences* 11(2): 41. DOI: https://doi.org/10.3390/admsci11020041.

Minuye B, Alebachew W, Kebede M, et al. (2021) Intention to care for COVID-19 patients among nurses working at health care institutions of Debre Tabor Town, North Central Ethiopia. *Risk Management and Healthcare Policy* Volume 14: 2475–2481. DOI: https://doi.org/10.2147/rmhp.s311830.

Moss J, Alexander L, Barré I, et al. (2023) Understanding physical distancing and face mask use across high-risk African American subgroups during the COVID-19 pandemic: application of health belief model. *Health Promotion Practice*: 152483992211511. DOI: https://doi.org/10.1177/15248399221151176.

Nahidi S, Sotomayor-Castillo C, Li C, et al. (2022) Australian critical care nurses' knowledge, preparedness, and experiences of managing

SARS-COV-2 and COVID-19 pandemic. *Australian Critical Care* 35(1): 22–27. DOI: https://doi.org/10.1016/j.aucc.2021.04.008.

Pesiridis T, Galanis P, Anagnostopoulou E, et al. (2021) Providing care to patients with COVID-19 in a reference hospital: health care staff intentional behavior and factors that affect it. *AIMS Public Health* 8(3): 456–466. DOI: https://doi.org/10.3934/publichealth.2021035.

Rahmatpour P, Sharif Nia H, Sivarajan Froelicher E, et al. (2020) Psychometric Evaluation of Persian Version of Nurses' Intention to Care Scale (P-NICS) for Patients with COVID-19. *International Journal of General Medicine* Volume 13: 515–522. DOI: https://doi.org/10.2147/ijgm.s260579.

Sharma L, Thapa A, Chand H, et al. (2020) Perception of doctors and nurses regarding prevention of COVID-19 infection at a Teaching Hospital, Nepal. *Journal of Patan Academy of Health Sciences* 7(3): 13–21. DOI: https://doi.org/10.3126/jpahs.v7i3.33820.

Storr-Mathis S (2021) *Barriers to effective personal protective equipment use in public health workers*. PhD Thesis, Walden University, UK. Available at: https://scholarworks.waldenu.edu/cgi/viewcontent.cgi?article=11819&context=dissertations (accessed 15 November 2022).

Tong L, Zhu M, Wang S, et al. (2022) Factors influencing caring behaviour among registered nurses during the COVID-19 pandemic in China: a qualitative study using the COM-B framework. *Journal of Nursing Management* 30(8). DOI: https://doi.org/10.1111/jonm.13855.

Zewude B, Melese B, Addis E, et al. (2021) Changing patterns of compliance with protective behavioral recommendations in the post first-round COVID-19 vaccine period among healthcare workers in Southern Ethiopia. *Risk Management and Healthcare Policy* 14: 3575–3587. DOI: https://doi.org/10.2147/rmhp.s325699.

Zhu P, Wu B, Tan J (Benjamin), et al. (2021) Nurses' willingness to participate in public health emergency: A qualitative study in China.

Precision Medical Sciences 10(4): 151–158. DOI: https://doi.org/10.1002/prm2.12052.

Supplementary File 4: Glossary

Terms	Definitions
Behaviours	Behaviour is an individual's actions or reactions to a situation, which can be voluntary or involuntary, conscious or unconscious (Taylor et al., 2007).
Theory	Theories are sets of statements or principles devised to explain a group of facts or phenomena that can be scientifically tested (Taylor et al., 2007: p.18).
Behavioural change Theory	Theories of change explain why behaviours change over time and how they can be changed (Darnton, 2008).
Model	Models are conceptual descriptions of a system, theory, or phenomenon that account for its known or inferred properties (Taylor et al., 2007: p.18).
Behaviour change models	Behavioural models help to understand particular behaviours by identifying the underlying factors that influence them. On the other hand, change models usually portray generic processes of change (Darnton, 2008).
Framework	A framework is an approach that is represented in a structured format composed of concepts and their interrelationships (Milton, 2010: p.1).
Behaviour change frameworks	The behaviour change framework (e.g., behaviour change wheel) provides a theoretical understanding of behaviour to determine what needs to change for the behavioural target to be achieved, and what intervention functions are likely to be effective to bring about that change (Michie et al., 2011: p.10).
Constructs	These are complex psychological and sociological concepts (defined as multi component theoretical concepts) such as attitudes, beliefs and subjective or descriptive norms contained in health behaviour change and other models (Taylor et al., 2007: p.18).
Health Belief model	A theoretical model for explaining and predicting individual changes in health

(HBM)<br>(Rosenstock, 1974)	behaviours.<br>*Key constructs include:*<br>Perceived susceptibility (individual's perceived threat to sickness or disease)<br>Perceived severity (belief of consequence)<br>Perceived benefits (potential positive benefits of action)<br>Perceived barriers (barriers to action)<br>Cues to action (exposure to factors that prompt action)<br>Self-efficacy (confidence in ability to succeed)<br>(LaMorte W., 2022a)
Theory of Planned behaviour (TPB) (Ajzen, 1991)	A behavioural change theory used to understand and predict behaviours which asserts that behaviours are determined by behavioural intentions and, in certain circumstances, perceived behavioural control.<br>*Key constructs include*:<br>Behavioural intention (motivational factors that influence behaviour)<br>Attitude (the degree to which a person has a favourable or unfavourable perception of a specific behaviour)<br>Subjective norm (social pressure to act or abstain from performing a specific behaviour)<br>Perceived behavioural control (perceptions of the difficulty or ease of executing the desired behaviour)<br>(Asare, 2015)
Trans-theoretical Model (TTM) (Prochaska and DiClemente, 1983)	A behavioural change model aimed at understanding individual behavioural changes and describing how people move dynamically through five different stages of behavioural change.<br>*Stages of change*<br>Precontemplation (lack of awareness that changing one's behaviour can improve one's life)<br>Contemplation (identification of the problem, preliminary consideration of behavioural change, and gathering information about potential solutions and actions).<br>Preparation (self-reflection on the decision, a reiteration of the need and desire to change behaviour, and completion of final pre-action steps)

	Action (putting in place, the practices needed for successful behaviour change)<br>Maintenance (strengthening of behaviours initiated during the action phase)<br>Termination (no desire to revert to the unhealthy former habits)<br>(LaMorte W., 2022b)
Protection Motivation Theory (PMT)<br>(Rogers, 1975)	A theory of behavioural change that describes the cognitive mediation process of behavioural change in terms of threat and coping appraisal.<br>*Components*<br>Threat appraisal: individuals' evaluation of the disease's severity (perceived severity) and estimation of the likelihood of contracting the disease (perceived vulnerability).<br>Coping appraisal: Individuals' expectation that following recommendations will eliminate the threat (response efficacy) and belief in one's ability to successfully carry out the recommended course of action (self-efficacy).<br>(Plotnikoff and Trinh, 2010)
Social-Cognitive Theory (SCT)<br>(Bandura, 1986)	A behavioural change theory which explains that learning takes place in a social context, with the person, environment, and behaviour interacting dynamically and reciprocally.<br>*Major constructs*<br>Personal factors (e.g., age, cognitions, previous experience with the behaviour)<br>Environmental factors (e.g., access to resources, safety, support from family/friends)<br>Aspects of the behaviour itself (e.g., vigour of the behaviour, outcomes achieved as a result of practicing the behaviour, competence with the behaviour).<br>(Mark and Paul, 2005)
Health Action Process Approach (HAPA)<br>(Schwarzer, 2008)	A model that explicitly includes post intentional mediators to overcome the intention-behaviour gap.<br>Phases<br>Motivation phase: pre-intentional motivation processes lead to a behavioural intention<br>Volition phase: post intentional volition processes lead to the actual health behaviour<br>(Schwarzer, 2008: p. 6)
Theoretical Domain Framework (TDF)<br>(Cane et al., 2012;	A theoretical framework that integrates components from 33 theories linked to behaviour change into 14 domains (original 12).<br>*14 domains*

Michie et al., 2005)	Knowledge; skills; social/professional role and identity; beliefs about capabilities; optimism; beliefs about consequences; reinforcement; intentions; goals; memory, attention, and decision processes; environment context and resources; social influences; emotion, and behavioural regulation. (Cane et al., 2012)
Capability, Opportunity and Motivation-Behaviour (COM-B) model (Michie et al., 2011)	A behaviour change model that is used to determine what needs to change for a behaviour change intervention to be effective. It identifies three elements that must exist for any behaviour to occur: capability, opportunity, and motivation.<br>*Elements*<br>Capability (an individual underlying psychological and physical abilities)<br>Opportunity (social and physical environments)<br>Motivation (automatic and reflective processes). (Michie et al., 2011)
Behaviour change wheel (Michie et al., 2011)	An integrated behavior change framework that consists of 19 different behavior change frameworks, with the COM-B model serving as its hub.<br>*Intervention Functions*<br>Education; persuasion; incentivization; coercion; training; restriction; environmental restructuring; modeling; enablement (Michie et al., 2011)

References for glossary

Ajzen I (1991) The Theory of Planned Behavior. *Organizational Behavior and Human Decision Processes* 50(2): 179–211.

Asare M (2015) Using the theory of planned behavior to determine the condom use behavior among college students. *American Journal of Health Studies* 30(1): 43–50. Available at: https://pubmed.ncbi.nlm.nih.gov/26512197/.

Bandura A (1986) *Social Foundations of Thought and Action*. Inc, Englewood Cliffs, NJ, US: Prentice Hall.

Cane J, O'Connor D and Michie S (2012) Validation of the theoretical domains framework for use in behaviour change and implementation research. *Implementation Science* 7(1). DOI: https://doi.org/10.1186/1748-5908-7-37.

Conner M and Norman P (2004) *Predicting Health Behaviour*. Maidenhead: Open University Press.

Darnton A (2008) *GSR behaviour change knowledge review reference report: an overview of behaviour change models and their uses*. July. Available at: https://assets.publishing.service.gov.uk/government/uploads/system/uploads/attachment_data/file/498065/Behaviour_change_reference_report_tcm6-9697.pdf.

LaMorte W (2022a) The health belief model. Available at: https://sphweb.bumc.bu.edu/otlt/MPH-Modules/SB/BehavioralChangeTheories/BehavioralChangeTheories2.html (accessed 11 November 2022).

LaMorte W (2022b) The transtheoretical model (stages of change). Boston University School of Public Health. Available at: https://sphweb.bumc.bu.edu/otlt/MPH-Modules/SB/BehavioralChangeTheories/BehavioralChangeTheories6.html (accessed 3 December 2022).

Michie S, Johnstone M, Abraham C, et al. (2005) Making psychological theory useful for implementing evidence based practice: a consensus approach. *Quality and Safety in Health Care* 14(1): 26–33. DOI: https://doi.org/10.1136/qshc.2004.011155.

Michie S, van Stralen MM and West R (2011) The behaviour change wheel: a new method for characterising and designing behaviour change

interventions. *Implementation Science* 6(42). DOI: https://doi.org/10.1186/1748-5908-6-42.

Milton NR (2010) A new framework for psychology. *Review of General Psychology* 14(1): 1–15. DOI: https://doi.org/10.1037/a0018325.

Plotnikoff RC and Trinh L (2010) Protection motivation theory. *Exercise and Sport Sciences Reviews* 38(2): 91–98. DOI: https://doi.org/10.1097/jes.0b013e3181d49612.

Prochaska JO and DiClemente CC (1983) Stages and processes of self-change of smoking: toward an integrative model of change. *Journal of Consulting and Clinical Psychology* 51(3): 390–395. DOI: https://psycnet.apa.org/doi/10.1037/0022-006X.51.3.390.

Rogers RW (1975) A protection motivation theory of fear appeals and attitude change. *The Journal of Psychology* 91(1): 93–114. DOI: https://doi.org/10.1080/00223980.1975.9915803.

Rosenstock IM (1974) The health belief model and preventive health behavior. *Health Education Monographs* 2(4): 354–386. DOI: https://doi.org/10.1177/109019817400200405.

Schwarzer R (2008) Modeling health behavior change: how to predict and modify the adoption and maintenance of health behaviors. *Applied Psychology* 57(1): 1–29. DOI: https://doi.org/10.1111/j.1464-0597.2007.00325.x.

Taylor D (2007) *Behaviour change: Taylor et al - models review*. Available at: https://www.nice.org.uk/guidance/ph6/resources/behaviour-change-taylor-et-al-models-review2 (accessed 1 December 2022).

Supplementary File 5: Additional results of individual studies (ordered alphabetically)

Citation of studies	Targeted IPC behaviours	Outcome measures	Results
Bashirian et al. (2020)<br><br>Iran	COVID-19 IPC behaviours Specified as: hand hygiene, gloves and masks use	Compliance and predictors	The HCWs were least compliant with gloves use during all procedures (43.3%) and face mask use at all times (51.8%). However, 87% and 84.6% of participants always cleaned their hands with water and soap and avoided interacting with others during their shift.
Castro-Sánchez et al. (2021)<br><br>UK	PPE use	Evaluation of PPE helper programme	The HCWs had concerns about at least one of the following aspects of PPE: adequacy and/or equity of supply across clinical areas and across professions, inconsistent advice and guidance, and level of training provided. The HCWs showed frustration, uncertainty, and anxiety in response to the frequent changes. They also reported a lack of trust in the PHE guidance, which occasionally reflected a loss of faith in leaders and managers about the supply of PPE.<br><br>The PPE Helper Program positively affected HCWs knowledge, attitudes, concerns, and behaviors towards PPE.
Curtis et al. (2022)<br><br>Australia	PPE use	Barriers to PPE use and evaluation of PPE intervention	PPE compliance varied between 47.9% (Buddy check) and 91.8% (Bare below elbow). Radiographers and medical students were the most compliant with PPE, followed by nurses, paramedics, and medical officers. Inpatient medical officers were the least compliant.<br><br>Direct observations made by a person designated to collect data on PPE use (PPE Marshal) revealed that the most common reasons for noncompliance with PPE regulations were a lack of access to a staff member who could serve as a buddy, depleted PPE stock at the bedside, general attire (e.g., wearing watches, long sleeves), and incorrect use of PPE.
Derksen et al. (2020)<br><br>Germany	Hand hygiene	Adherence and determinants	Adherence to hand hygiene recommendations increased from 47% prior to the COVID-19 pandemic to 95% just before the lockdown while simple pandemic measures were implemented.<br><br>Only self-efficacy and gender were significant predictors of hand hygiene behaviour in the regression model when hospital, time period, age, and profession were controlled for.<br><br>Age was also significantly associated with hand hygiene in the mediation model.
Jeong and Eun (2023)<br><br>Korea	COVID-19 IPC behaviours (Unspecified)	Levels and associated factors	The mean score for infection control practices related to COVID-19 was 4.76 on a 5-point scale where a higher score indicated superior infection control performance. Multiple regression analysis revealed that the factors that influenced COVID-19 infection control practices were gender, marital status, perceived susceptibility, and confidence in practice related to COVID-19.
Kim and Kim	COVID-19 IPC behaviours	Associated factors	The participants' mean score for COVID-19-related infection control practices was 4.60 out of 5; for knowledge about COVID-19, 19.53 out of 25; for health beliefs related to COVID-19, 3.96 out of 5. The level of COVID-19-related

Citation of studies	Targeted IPC behaviours	Outcome measures	Results
(2023)<br>Korea	(unspecified)		infection control practices was significantly higher in nurses who had received education about emerging infectious diseases and in those who had been monitored for wearing PPE compared to those who had not.
Limkunakul et al. (2022)<br>Thailand	COVID-19 IPC behaviours Specified as: hand hygiene, surgical masks, disinfection	Predictors	The preventive behaviour category had an average score of 87.6% (70.3/80). After adjusted, knowledge and perception of personal preventability were independently associated with preventive behaviours. The adjusted coefficients of both factors were- 0.911 (p= 0.009) and 0.477 ($p < 0.001$).
Lohiniva et al. (2022)<br>Finland	COVID-19 IPC behaviours Specified as: hand hygiene, PPE, environmental cleaning, other	Compliance and factors	Most HCWs (83.2%) had difficulty complying with at least one COVID-19-specific IPC measure (hand hygiene, PPE, environmental cleaning, other).<br>Over one-third (36.5%) found it challenging to use PPE, and about one-quarter (25.6%) had difficulty with environmental cleaning. However, only 3.6% of HCWs reported a problem with hand hygiene procedures.<br>Other issues were reported by 29.1% of HCWs, with the most common being related to visiting policies (33.6%).
Mortada et al. (2021)<br>Saudi Arab	COVID-19 IPC behaviours (Unspecified)	Behavioural intention and determinants	A majority (85.7%) of HCWs, had the intention to comply with COVID-19 preventive behavior. Being female, being a nurse, being IPC methods, and having access to PPE while at work were significantly associated with intention to comply with COVID-19 preventive behavior ($p \leq 0.01$).
Müller et al. (2021)<br>Germany	COVID-19 IPC behaviours Specified as: PPE, hand hygiene, disinfection	Barriers and enablers	All dentists (n=12) had infection control measures in place. FFP2 masks, face shields (impractical), team rotation (inadequate staffing), and avoidance of aerosol-generating treatments were the most frequently ignored measures.<br>Dentists with COVID-19 experience or who saw themselves as role models were more eager to implement measures.
Salwa et al. (2022)<br>Bangladesh	COVID-19 IPC behaviours Specified as: disinfection, decontamination,	Compliance and associated factors	The HCWs' compliance with WHO-recommended IPC guidance was unsatisfactory (mean compliance score of 0.49 (±0.25) on a 0–1 scale). The HCWs were most compliant with the medical mask-wearing guidelines (81%) and were least compliant with the high-touch surface decontamination regulations (23%). Compliance with the IPC guidance was significantly associated with increasing age, female sex, working as a nurse, having non-communicable diseases, and history of exposure to patients with COVID-19 ($p<0.001$)

Citation of studies	Targeted IPC behaviours	Outcome measures	Results
	hand hygiene, PPE use		
Seitz et al. (2021)<br><br>USA	PPE use	Knowledge, attitude and practice	Self-reported compliance with PPE use was high (96%). All HCWs had sufficient knowledge of PPE use. Approximately half (47.6%) felt that PPE was inconvenient and 28.2% reported that PPE interfered with patient care.
Silverberg et al. (2021)<br><br>Canada	COVID-19 IPC behaviours Specified as: hand hygiene, use of mask, gloves, gowns and face shields, PPE use	Adherence and associated barriers and facilitators	The HCWs reported a high rate of PPE use for suspected or confirmed COVID-19 cases, with 100% coverage for hand hygiene and high rates of use of face masks (97%) and disposable gloves (99%). Additionally, the respondents reported frequent use of gowns and face shields (86% each).
Sin and Rochelle (2022)<br><br>Hong Kong	Hand hygiene	Beliefs and behaviours	Self-reported hand hygiene performance was high (81.93%) among nurses.
Sivaraman et al. (2022)<br><br>India	PPE use	Behavioural intention	PPE use among otorhinolaryngology HCWs was influenced by various aspects of the HBM, including perceived susceptibility, perceived severity of the disease, perceived barriers and benefits in PPE use, self-efficacy, health-related knowledge, and cues to action.
Toghanian et al. (2022)<br><br>Iran	COVID-19 IPC behaviours (Unspecified)	Related factors	The mean score for HCWs' protective behaviours was 4.20 (SD = 0.56), and it was significantly higher in nurses, women, married people, and those with a BSc degree than in others ($p < 0.05$).
Türktemiz et al. (2021)<br><br>Turkey	PPE use	Perceptions and attitude	Most (96.20%) of the HCWs considered personal protective equipment beneficial and a wise preventive measure. However, HCWs expressed challenges to PPE use: difficulty in providing patient care (76.6%) and discomfort related to its use (82.30%).

Citation of studies	Targeted IPC behaviours	Outcome measures	Results
van Hout et al. (2022)<br><br>Europe	COVID-19 IPC behaviours<br>Specified as: hand hygiene and PPE use	Perceptions and compliance	Most HCWs (79%) had positive perceptions towards the protective effects of IPC measures. More than 80% of the respondents had self-reported compliance to WHO recommended IPC behavior (hand hygiene, gloves, face mask, N95 respirator use, eye protection, fluid-resistant gowns) with both aerosol-generating and non-aerosol-generating procedures.
Yang et al. (2021)<br><br>China	COVID-19 IPC behaviours<br>Specified as: hand hygiene, overall droplet isolation, google and gown use	Levels and determinants	The HCWs reported good IPC practices during the COVID-19 pandemic, with relatively high compliance with hand washing (96.37%) and the use of masks (97.94%), gloves (95.82%), gown (85.52%).<br><br>HCWs in high-risk departments showed better behaviour of gown use ($\beta = 0.158$, $p = 0.032$), while HCWs who had contact with confirmed or suspected patients had worse behaviours of goggle ($\beta = -0.127$, $p = 0.050$) and gown use ($\beta = -0.153$, $p = 0.003$).<br><br>HCWs with higher education degree reported low compliance of gown use ($\beta = -0.080$, $p = 0.003$).

BCW: behavior change wheel; CI: Confidence Interval; COM-B: capability, opportunity, motivation and behavior; FFP: filtering face piece; HBM: health belief model; HCWs: health care workers; HAPA: health action process approach; IPC: infection prevention and control; PMT: protection motivation theory; PPE: personal protective equipment; SD: standard deviation; TDF: theoretical domain framework; TPB: theory of planned behavior; TTM: trans-theoretical model; WHO: World Health Organization

Supplementary File 6: Quality appraisal of included studies using the MMAT checklist

Studies	1.1	1.2	1.3	1.4	1.5	4.1	4.2	4.3	4.4	4.5	5.1	5.2	5.3	5.4	5.5	Quality
Bashirian et al. (2020)						1	1	1	1	1						*****
Castro-Sánchez et al. (2021)						1	1	1	1	1						*****
Curtis et al. (2022)	1	0	1	1	1											****
Derksen et al. (2020)						1	0	1	0	1						***
Jeong and Eun (2023)						1	1	1	1	1						*****
Kim and Kim (2023)						1	1	1	1	1						*****
Limkunakul et al. (2022)						1	1	1	1	1						*****
Lohiniva et al. (2022)	1	1	1	1	1	1	0	1	0	1	1	1	1	1	1	***
Mortada et al. (2021)						1	1	1	1	1						*****
Müller et al. (2021)	1	1	1	1	1											*****

Salwa et al. (2022)#	-	-	-	-	-	1	1	1	1	1	-	-	-	-	-	-
Seitz et al. (2021)						1	1	0	1	1						****
Silverberg et al. (2021)						1	0	1	0	1						***
Sin and Rochelle (2022)						1	1	1	0	1						****
Sivaraman et al. (2022)	1	1	1	1	1											*****
Toghanian et al. (2022)						1	1	1	1	1						*****
Türktemiz et al. (2021)						1	1	0	0	1						***
van Hout et al. (2022)						1	1	0	0	1						***
Yang et al. (2021)						1	1	1	0	1						****

Only quantitative findings of this study have been reported

1.1. Is the qualitative approach appropriate to answer the research question? 1.2. Are the qualitative data collection methods adequate to address the research question? 1.3. Are the findings adequately derived from the data? 1.4. Is the interpretation of results sufficiently substantiated by data? 1.5. Is there coherence between qualitative data sources, collection, analysis and interpretation? (MMAT Criteria - Qualitative studies)

4.1. Is the sampling strategy relevant to address the research question? 4.2. Is the sample representative of the target population? 4.3. Are the measurements appropriate? 4.4. Is the risk of non- response bias low? 4.5. Is the statistical analysis appropriate to answer the research question? (MMAT Criteria- Quantitative studies)

5.1. Is there an adequate rationale for using a mixed- methods design to address the research question? 5.2. Are the different components of the study effectively integrated to answer the research question? 5.3. Are the outputs of the integration of qualitative and quantitative components adequately interpreted? 5.4. Are divergences and inconsistencies between quantitative and qualitative results adequately addressed? 5.5. Do the different components of the study adhere to the quality criteria of each tradition of the methods involved? (MMAT Criteria-Mixed method)

Rating: 5*****: 100% quality criteria met; 4 ****: 80% quality criteria met; 3 ***: 60% quality criteria met; 2 **:40% quality criteria met; 1*: 20% quality criteria met
